# Effect of Pre-weaning Diet on the Ruminal Archaeal, Bacterial, and Fungal Communities of Dairy Calves

**DOI:** 10.3389/fmicb.2017.01553

**Published:** 2017-08-15

**Authors:** Juliana Dias, Marcos I. Marcondes, Melline F. Noronha, Rafael T. Resende, Fernanda S. Machado, Hilário C. Mantovani, Kimberly A. Dill-McFarland, Garret Suen

**Affiliations:** ^1^Department of Animal Science, Universidade Federal de Viçosa Viçosa, Brazil; ^2^Coordenação de Aperfeiçoamento de Pessoal de Nível Superior, Ministério da Educação Brasília, Brazil; ^3^Division of Microbial Resources, Research Centre for Chemistry, Biology and Agriculture, University of Campinas Campinas, Brazil; ^4^Forestry Department, Universidade Federal de Viçosa Viçosa, Brazil; ^5^Embrapa Dairy Cattle Juiz de Fora, Brazil; ^6^Department of Microbiology, Universidade Federal de Viçosa Viçosa, Brazil; ^7^Department of Bacteriology, University of Wisconsin-Madison Madison, WI, United States

**Keywords:** microbiota, archaea, bacteria, fungi, rumen, dairy calves, diet, age

## Abstract

At birth, calves display an underdeveloped rumen that eventually matures into a fully functional rumen as a result of solid food intake and microbial activity. However, little is known regarding the gradual impact of pre-weaning diet on the establishment of the rumen microbiota. Here, we employed next-generation sequencing to investigate the effects of the inclusion of starter concentrate (M: milk-fed vs. MC: milk plus starter concentrate fed) on archaeal, bacterial and anaerobic fungal communities in the rumens of 45 crossbred dairy calves across pre-weaning development (7, 28, 49, and 63 days). Our results show that archaeal, bacterial, and fungal taxa commonly found in the mature rumen were already established in the rumens of calves at 7 days old, regardless of diet. This confirms that microbiota colonization occurs in the absence of solid substrate. However, diet did significantly impact some microbial taxa. In the bacterial community, feeding starter concentrate promoted greater diversity of bacterial taxa known to degrade readily fermentable carbohydrates in the rumen (e.g., *Megasphaera, Sharpea*, and *Succinivribrio*). Shifts in the ruminal bacterial community also correlated to changes in fermentation patterns that favored the colonization of *Methanosphaera* sp. A4 in the rumen of MC calves. In contrast, M calves displayed a bacterial community dominated by taxa able to utilize milk nutrients (e.g., *Lactobacillus, Bacteroides*, and *Parabacteroides*). In both diet groups, the dominance of these milk-associated taxa decreased with age, suggesting that diet and age simultaneously drive changes in the structure and abundance of bacterial communities in the developing rumen. Changes in the composition and abundance of archaeal communities were attributed exclusively to diet, with more highly abundant *Methanosphaera* and less abundant *Methanobrevibacter* in MC calves. Finally, the fungal community was dominated by members of the genus SK3 and *Caecomyces*. Relative anaerobic fungal abundances did not change significantly in response to diet or age, likely due to high inter-animal variation and the low fiber content of starter concentrate. This study provides new insights into the colonization of archaea, bacteria, and anaerobic fungi communities in pre-ruminant calves that may be useful in designing strategies to promote colonization of target communities to improve functional development.

## Introduction

In adult ruminants, the rumen harbors a diverse microbiota composed of archaea, bacteria, fungi, and ciliated protozoa species that act synergistically to degrade feedstuffs and provide nutrients such as, volatile fatty acids (VFAs), protein, minerals, and vitamins to the host (Hungate, 1966). However, young ruminants have an immature gastrointestinal tract (GIT) and are thought to be sterile at birth (Taschuk and Griebel, 2012). Therefore, the rumen's contributions to nutrient degradation and the provision of energy are minimal in the pre-ruminant calf, relative to more advanced stages of development.

Until the third week of life, calves are considered non-ruminants, because their rumen is anatomically and physiologically underdeveloped (Davis and Drackley, 1998; Baldwin et al., 2004). At this stage, their diet is predominantly milk, which bypasses the rumen and is carried by the esophagical groove into the fourth stomach, termed the abomasum (Davis and Drackley, 1998). The beginning of solid food intake (i.e., starter concentrate) around 3 weeks of age triggers a critical process of transition from a functional non-ruminant to a true ruminant that relies on the establishment and activity of the rumen microbiota. During this transition stage (3–8 weeks), solid intake increases, and this increasing supply of substrates allows for microbial degradation, resulting in increased VFA concentrations within the rumen. These events promote a cascade of morpho-physiological shifts (i.e., rumen papillation and increased volume) in the digestive system that result in a fully functional rumen at weaning (Warner et al., 1956; Davis and Drackley, 1998).

Given the critical nature of the rumen microbiota in developing calves, surprisingly little is known about the establishment and changes in composition of the rumen microbiota during the pre-weaning period. Because bacteria constitute a predominant and diverse GIT microbial domain that plays several roles, including modulation of the immune system (Bauer et al., 2006) and metabolism of nutrients (Hungate, 1966), most research has focused on the bacterial community (Li et al., 2012; Wu et al., 2012; Jami et al., 2013; Rey et al., 2014). This provides a restricted picture of the microbial diversity that exists within the calf rumen.

In addition to bacteria, methanogenic archaea are also known to colonize the GIT shortly after birth (Gagen et al., 2012; Guzman et al., 2015) and play an important ecological role in the mature rumen by continuous removal of hydrogen gas (H_2_), which may constrain microbial growth and carbohydrate degradation at high levels (Wolin et al., 1997). However, ruminal archaea also synthesize methane (CH_4_), which is a potent greenhouse gas released during eructation and a relevant source of dietary energy loss for the host (Liu and Whitman, 2008; Knapp et al., 2014). Given that manipulation of the archaeal community is desirable for both environmental and nutritional aspects, the identification of the factors that affect its establishment in the developing rumen may guide efforts in the formulation of strategies to mitigate enteric methane emissions with long-term effects and economic viability.

Anaerobic fungi, like the ruminal bacteria, are known to play a prominent role in the degradation of fibrous plant material due to their ability to disrupt vegetal tissues and facilitate the colonization of fiber particles by fibrolytic bacteria. The ruminal fungi also produce a wide range of polysaccharide-hydrolyzing enzymes to ferment complex carbohydrates, releasing great amounts of H_2_ that favor the archaeal community (Bauchop, 1983; McAllister et al., 1993). Therefore, anaerobic fungal activity may shape the structure of the bacterial and archaeal communities in the mature rumen, and consequently, influence fiber utilization efficiency and methanogenesis (Tripathi et al., 2007; Kittelmann et al., 2012). Some anaerobic fungal communities are able to establish in the rumen even in the absence of dietary fiber, as observed in lambs (Fonty et al., 1987), but the factors that affect their establishment, distribution and survival in the developing bovine rumen remains poorly explored.

Like the anaerobic fungi, ciliate protozoa have a close association with rumen archaeal and bacterial communities and can impact nutrient digestibility, fermentation and methanogenesis (Newbold et al., 2015). However, unlike other microbial groups, the establishment of ciliate protozoa in the rumen of young ruminants is reliant on direct or indirect contact with the saliva of adult animals (Coleman, 1979). In contrast to beef cattle production system where calves experience maximum maternal care, dairy calves are separated from their dams right after birth and kept isolated until weaning. Under these conditions, dairy calves are naturally protozoa-free (Sahoo et al., 2005).

Overall, information related to the archaeal and fungal communities in the developing rumen is limited to a handful of studies (Fonty et al., 1987; Gagen et al., 2012; Zhou et al., 2014; Guzman et al., 2015) with only one study investigating all three microbial domains in calves concurrently (Dill-McFarland et al., 2017). Previous work reported that bacterial communities change with age (Jami et al., 2013; Rey et al., 2014; Dill-McFarland et al., 2017) but no sequencing-based analysis has been done to concurrently assess archaeal, bacterial and fungal communities in response to pre-weaning diets.

The identification of intra- and inter-communities co-occurrence and interactions may guide the elaboration of strategies to promote colonization of microbial groups linked to health and performance of dairy calves, especially bred in challenging systems. Lastly, since the ruminant microbiota is resilient to change once it is established (Weimer, 2015), it is important to track changes in the microbiota of young calves in order to determine the most suitable window of time for microbial interventions.

Here, we hypothesized that diet composition impact the establishment of the rumen microbiota by selecting for taxa adapted to dietary substrates and that the ruminal microbial community changes with age by responding to the increase of solid feed intake, leading to increased microbial fermentation and rumen metabolic development. As such, the objective of this study was to characterize changes in the rumen archaeal, bacterial, and fungal communities of Holstein-Gyr crossbred dairy calves across pre-weaning development on two different diets.

## Materials and methods

### Animals

All animal procedures were conducted according to protocols approved by the Universidade Federal de Viçosa (Minas Gerais, Brazil) Animal Care and Use Committee, protocol number 27/2013. The study was carried out at the Experimental Field of Embrapa Dairy Cattle, located in Coronel Pacheco, Minas Gerais, from October to April 2014. A total of 45 newborn male crossbred (3/4 to 15/16 Holstein × Gyr) dairy calves from a fixed-time insemination protocol were removed from their dams after birth (within 24 h), weighed (35 ± 3.6 kg), identified and housed in individual shelters to avoid direct contact between calves and mature animals. Colostrum from their dams was offered to 10% of body weight at birth (BW), fractionated into two daily meals (morning and afternoon) until the 3rd day of life. Calves were randomly assigned to one of two diets (M: only whole milk to 10% of BW or MC: whole milk to 10% of BW plus starter concentrate *ad libitum*) and four slaughter age groups (7, 28, 49, and 63 days). Thus, 7 groups were formed: M-7d (*n* = 6), M-28d (*n* = 6), M-49d (*n* = 7), M-63d (*n* = 6), MC-28d (*n* = 6), MC-49d (*n* = 8) and MC-63d (*n* = 6). Regardless of diet group, water was available to all animals *ad libitum*. Calves slaughtered at 7 days of age were fed exclusively colostrum and milk, because starter concentrate intake is negligible in the first week of life. The starter concentrate was formulated to provide National Research Council (2001) dietary recommendations for dairy calves, and was made available in buckets, which were refilled daily after evaluating intake (Supplementary Table 1).

### Sample collection

Calves were euthanized with an injection of Acepromazine (0.013 mg/kg), Thiopental (0.125 mg/kg), and Potassium chloride (80–120 mL). Immediately after euthanasia, the body cavity was opened and the rumen was isolated with polyethylene seals (zip locks) to avoid reflux of ingesta between compartments. Ruminal aliquots (50 and 25 mL) were collected and stored at −80°C until DNA extraction and VFA analysis.

### Volatile fatty acid analysis

The VFA concentration of rumen samples was determined using high-performance liquid chromatography (HPLC). In brief, the samples of ruminal fluid were thawed at room temperature, centrifuged (12,000 × g for 10 min at 4°C) and the supernatant (1.5 mL) was treated using the methods described by Siegfried et al. (1984) and analyzed by HPLC using a Dionex Ultimate 3,000 Dual system with a Shodex RI-101 refractive index detector and a Phenomenex Rezex ROA (300 × 7.8 mm) ion-exclusion column at 45°C. The mobile phase was analyzed using H2SO4 (5 mM) at a flow of 0.7 mL/min. Samples were compared to standards containing increasing concentrations (up to 20 mM) of acetate, butyrate, propionate, formate, isobutyrate, isovalerate, succinate, valerate, and lactate.

### DNA extraction and sequencing

Total genomic DNA was extracted from rumen fluid following methods described by Stevenson and Weimer (2007). Briefly, the samples of ruminal fluid were thawed at room temperature, centrifuged (8,000 × g for 25 min at 4°C) and the supernatant was discharged. The cells were re-suspended in 2 mL of cold extraction buffer and 1 mL of cell suspension was lysed by heating and mechanical disruption. DNA was purified by phenol and phenol:chloroform:isoamyl alcohol extraction and resuspended in TE buffer. DNA was quantified using a Nanodrop spectrophotometer (Thermo Scientific, Wilmington, DE) and shipped on dry ice to the University of Wisconsin-Madison for sequencing. The V3-V4 hypervariable regions of the bacterial 16S rRNA, V6-V8 of the archaeal 16S rRNA and the fungal internal transcribed spacer (ITS1) were amplified using primers described by Klindworth et al. (2013) and Kittelmann et al. (2013). For bacteria, PCR reactions consisted of 50 ng template DNA, 0.4 μM of each primer, 1X Kapa Hifi HotStart ReadyMix (KAPA Biosystems), and water to 25 μL. For archaea and fungi, DNA was increased to 100 ng and primers to 1.6 μM each. PCR was performed at 95°C for 3 min, 95°C for 30 s, 55°C for 30 s, 72°C for 30 s (25 cycles for bacteria; 35 cycles for archaea and fungi) and a final extension step at 72°C for 5 min. PCR products were purified by PureLink® Pro 96 PCR Purification Kit (Invitrogen) and a second PCR was performed on products to attach Illumina sequencing adapters and unique dual indices. PCR reactions were similar to those for V3–V4 (bacteria) except 5 μL of unquantified PCR product was used as template DNA and 8 cycles were performed. PCR products were recovered by gel extraction in AquaPōr LM low-melt agarose (National Diagnostics, Atlanta, GA) using the Zymoclean Gel DNA Recovery Kit (Zymo Research, Irvine, CA). Purified DNA was quantified by Qubit® Fluorometer (Invitrogen) and equimolar pooled to create a single sample at 1 × 10^9^ ng per μL). Sequencing was performed using the 2 × 300 bp paired-end method on an Illumina MiSeq following manufacturer's guidelines (Illumina, Inc., San Diego, CA, USA). All DNA sequences have been deposited in the NCBI's Short Read Archive under BioProject PRJNA381944.

### Bioinformatics analysis

Archaea, bacteria and fungi sequences were processed separately using mothur (v1.35.0) (Schloss et al., 2009) with procedures modified from Kozich et al. (2013). Briefly, paired-end reads were joined using default parameters in make.contigs (same base bonus = 1, different base penalty = −1, gap penalty = −2, gap extension penalty = −1). Sequences with a length shorter than 250 bp or longer than 600 bp containing ambiguous characters or exhibiting a homopolymer greater than 8 bp were removed. Archaeal and bacterial sequences were aligned using the SILVA 16S rRNA gene reference database (Pruesse et al., 2007) and pre-clustered to remove sequencing errors. Fungal sequences were de novo aligned and pre-clustered. The Uchime algorithm was used to detect chimeric sequences (Edgar et al., 2011) and sequences that did not align to the correct region or were chimeric were removed. The sequences were taxonomy assigned using the Wang method, with a bootstrap cut-off of 80. Taxonomic assignment of archaeal, bacterial, and fungal ITS sequences was performed using respectively, the RIM-DB (Seedorf et al., 2014), Greengenes (DeSantis et al., 2006), and Neocallimastigomycota (Koetschan et al., 2014) reference databases. All sequences were grouped into operational taxonomic units (OTUs) by uncorrected pairwise distances clustered by the nearest neighbor method with a similarity cutoff of 97%. Coverage was assessed by Good's coverage and samples that displayed coverage <95% (two samples in the fungi data set) were discarded prior to normalization. Due to different sequencing depths, OTU tables were normalized to equal sequence counts, with 103 archaeal, 3,012 bacterial, and 107 fungal sequences per sample. The normalized OTU tables were used to determine the alpha diversity (Chao1, Shannon and inverse Simpson) indexes and the relative abundance (reads/total reads in a sample) of OTUs and higher taxa in each rumen sample.

### Statistical analysis

All statistical analyses were performed in R (v3.2.3) and tests were assessed as significant if *P*-values and or False Discovery Rate (FDR) ≤ 0.05. Analysis of calf genetic group showed non-significant effects (*P* > 0.05) and were not included in the models used here (Supplementary Table 2). Differences in archaeal, bacterial, and fungal alpha-diversity, as determined by Chao1, inverse Simpson's and Shannon's diversity indices as well as VFA concentration of rumen samples in response to diet, age, and interaction (diet^*^age), were assessed by a two-way ANOVA (type III error). The *P*-values were adjusted for FDR using the Benjamini-Hochberg method. This analysis was performed in R using functions available in the car package (Fox and Weisberg, 2011).

Beta-diversity of archaeal, bacterial, and fungal communities were determined by two approaches: Venn diagrams to visualize how many unique OTUs are shared across developmental stages of M and MC calves, as well as canonical analysis of principal coordinates (CAP) to assess dissimilarities in the microbial community composition. Venn diagrams were built with OTUs that represented ≥0.1% of the total community within each of the microbial groups (bacteria, archaea or fungi) in at least one sample and that were detected in at least two calves in each diet^*^age group using functions available in the R packages VennDiagram (Chen, 2015) and venn (Dusa, 2016). The CAP analysis was performed with OTUs (at >0.1% relative abundance in at least one sample) clustered by the Bray-Curtis dissimilarity index corrected according to Legendre and Legendre (1998). The permutation test (nperm = 999) was performed to assess the significance of constraints for each factor (diet, age, and diet^*^age) as well as for each constrained axis (CAP1 and CAP2). The Bray-Curtis dissimilarities ordinated by CAP were plotted according to diet and age groups with ellipses defined by standard deviation with a 95% confidence limit. These analyses were performed in R using functions available in vegan (Oksanen et al., 2013) and ggplot2 (Wickham, 2009).

Differences in the relative abundance of archaeal, bacterial, and fungal taxa in response to diet, age, and diet^*^age were assessed by Poisson regression (Jonsson et al., 2016), followed by analysis of deviance (*F*-test, type III error) and Tukey's Honest Significant Difference test (HSD). The *P*-values were adjusted for FDR using the Benjamini-Hochberg method. This analysis was performed using functions available in the packages car and agricolae in R (De Mendiburu, 2015). Only genera that represented ≥0.1% of the total community within each of the microbial groups (bacteria, archaea, or fungi) in at least one sample and that were detected in at least 50% of all samples were included.

The inter- and intra-genera association patterns of archaeal, bacterial, and fungal communities were assessed by two approaches: the Dice index to measure the ecological distance among taxa indicating their co-occurrence (Dice, 1945) and Spearman's rank correlation, which indicates the strength of association among taxa. Spearman's rank correlation was also employed to assess the relationship between molar proportions of VFAs (acetate, butyrate, propionate, acetate-to-propionate ratio, and total VFA) and relative abundance of archaeal, bacterial, and fungal taxa. Only taxa that represented ≥0.5% of the total community in at least one sample within each of the microbial groups (bacteria, archaea, or fungi) and that were detected in at least 50% of all samples were included. These analysis were performed in R across all samples of calves grouped according to diet using functions available in the packages arules (Hahsler et al., 2005), Hmisc (Harrell, 2016), and corrplot (Wei, 2013).

## Results

### Sequencing

After sequence trimming, quality filtering, 84,280 (mean 2,159.0 ± SD 1,872.9 per sample) high-quality archaeal, 979,883 (21,775.2 ± 21,211.5) bacterial, and 32,099 (923.8 ± 746.5) fungal sequences were obtained. The Good's coverage estimator ranged from 0.96 to 1.0, indicating that sequences sufficiently covered the diversity of archaeal, bacterial, and fungal communities in all samples. A summary of chimera identification, number of sequences and OTU numbers prior to and after normalization according to microbial domain, diet, and age group is shown in Supplementary Tables 3, 4.

### Archaeal populations vary with diet but not age

For our archaeal alpha-diversity analysis, Chao1 richness, inverse Simpson's diversity and Shannon's diversity, respectively, did not differ by diet (ANOVA, *P* = 0.608, 0.328, 0.476), age (*P* = 0.608; 0.608, 0.608) or the interaction of these two factors (*P* = 0.646; 0.608; 0.646; Supplementary Table 5).

**Figure 1:**
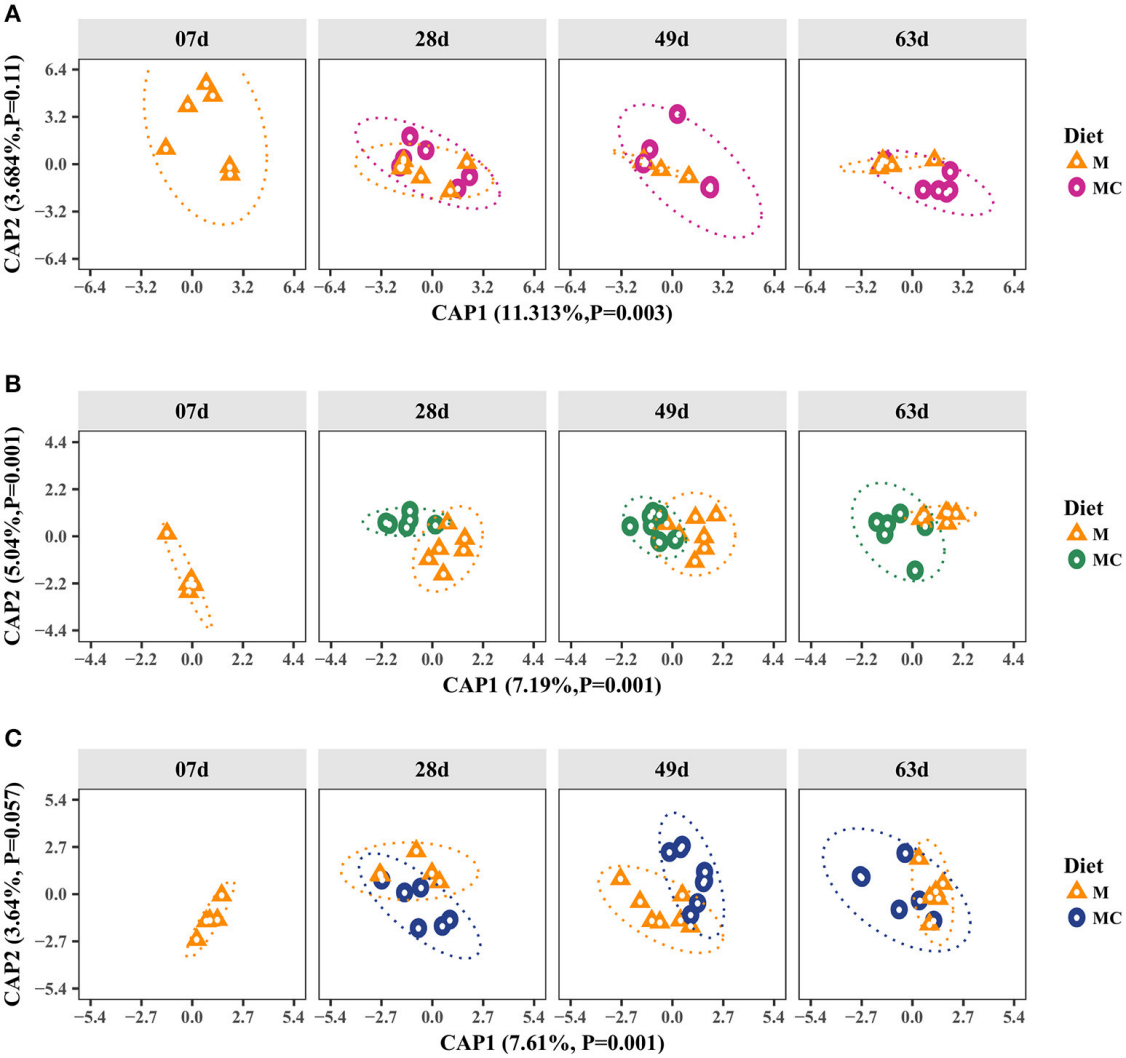
Canonical Analysis of Principal Coordinates (CAP) of the Bray-Curtis dissimilarity metric for archaeal **(A)**, bacterial **(B)**, and fungal **(C)** communities in the rumen of dairy calves. Individual points in each plot represents a rumen sample, different shapes represent diet (M: milk-fed or MC: milk and starter concentrate fed) and each facet represents the age group (7, 28, 49, and 63 days old). Percentages and *P*-values shown along the axes represent, respectively, the proportion of dissimilarities captured by CAP and significance of Permutation test for each axis. The increasing distance between samples equates to more dissimilarity in the community composition.

Beta-diversity analysis showed that Bray-Curtis dissimilarities in the archaeal community were significantly different according to diet (Permutation test, *P* = 0.009). This diet effect (i.e., inclusion of starter concentrate) was more evident among M and MC calves at 49 and 63 days, which clustered separately (Figure 1A). However, these shifts were not significantly ascribed to age group or interaction (*P* = 0.393; 0.305). Our Venn diagram analysis showed that out of 7 archaeal OTUs (at >0.1% relative abundance in at least one sample) only 3 were shared across developmental stages (7, 28, 49, and 63 days old) of M or MC calves (Supplementary Figure 1). However, in both diet groups, the number of shared OTUs (*n* = 5) did not change among calves at 28, 49, and 63 days old, indicating that archaeal community composition of calves at 7 days old is distinct from older calves (Supplementary Figure 1).

**Figure 2:**
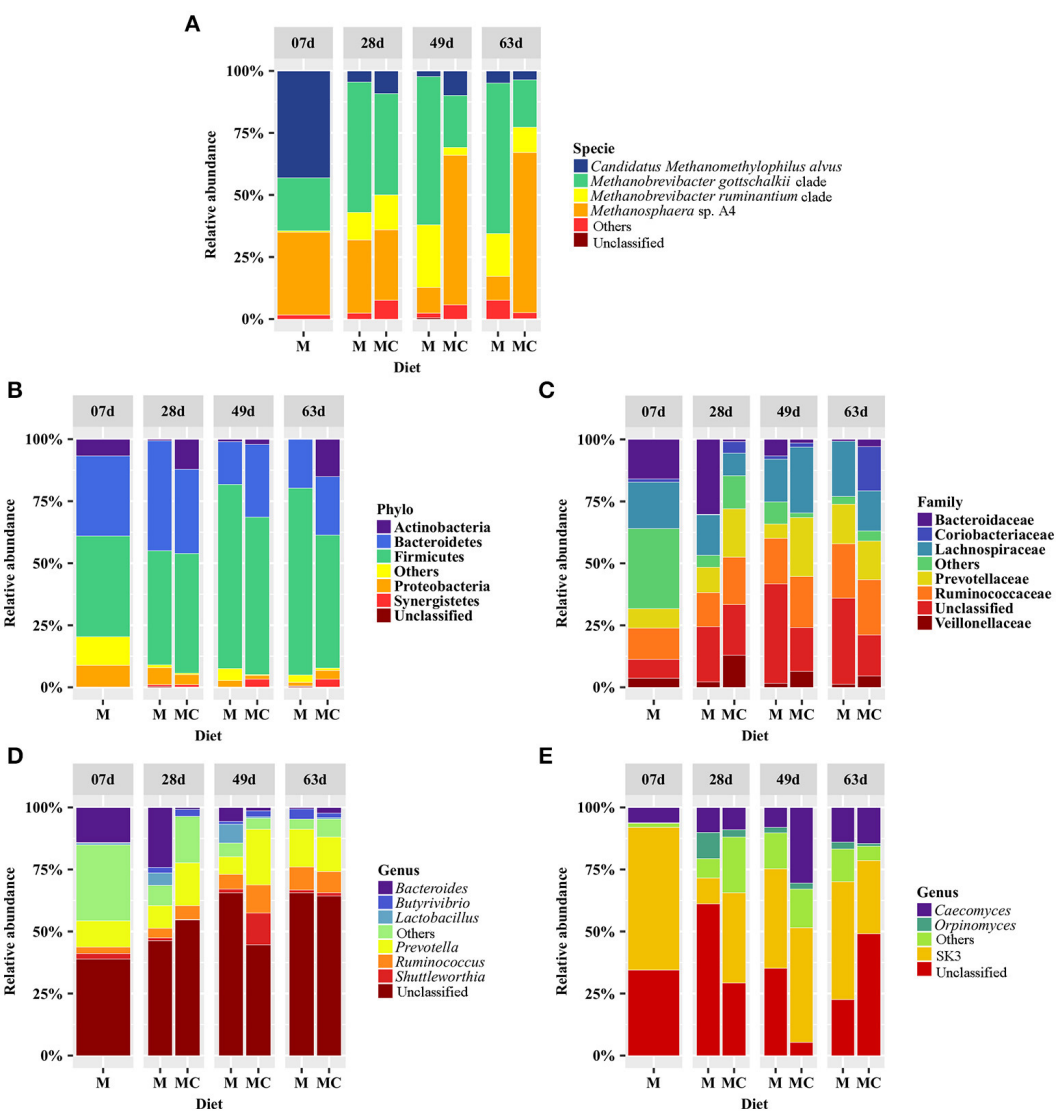
Distribution of the most abundant archaeal **(A)**, bacterial **(B–D)**, and fungal **(E)** taxa with average abundance ≥1% and that were identified in at least 50% of all samples in the rumen of calves grouped according to diet (M: milk-fed or MC: milk and starter concentrate fed) and age group (7, 28, 49, and 63 days). The group “Others” refers to taxa that displayed relative abundances <1.0% whereas “Unclassified” refers to sequences that could not be assigned to phylum, family, genus, or species level.

Taxonomic composition analysis of the archaeal community revealed 9 OTUs (mean 3.2 ± SD 1.1) annotated to the phyla Euryarchaeota (99.9 ± 0.1%) and Crenarchaeota (0.1 ± 0.1%) including the families Methanobacteriaceae (89.1 ± 3.3%), Methanomassiliicoccaceae (10.8 ± 3.3%), and Sulfolobaceae (0.1 ± 0.1%) as well as the genera *Methanobrevibacter* (53.3 ± 6.3%), *Methanosphaera* (35.7 ± 5.9%), Methanomassiliicoccaceae Group 11 (10.8 ± 3.3%), *Sulfolobus* (0.1 ± 0.1%), and Methanomassiliicoccaceae Group 9 (<0.1 ± 0.1%). At the species level, *Methanobrevibacter gottschalkii* (39.0 ± 6.2%), *Methanosphaera* sp. A4 (34.4 ± 6.1%), *Methanobrevibacter ruminantium* (11.6 ± 2.7%) and *Candidatus Methanomethylophilus alvus* (10.8 ± 3.3%) dominated the archaeal community while *Methanobrevibacter boviskoreani* (2.7 ± 1.0), *Methanosphaera* sp. ISO3-F5 (1.3 ± 0.4), *Sulfolobus thuringiensis* (0.1 ± 0.1%) and Methanomassiliicoccaceae Group 9 sp. ISO4-G1 (<0.1 ± 0.1%) were identified in <50% of all samples. The distributions of archaeal taxa among individual calves and diet-age groups are displayed in Supplementary Figure 2 and Figure 2, respectively.

Our analysis of deviance showed that the relative abundances of members of the genera *Methanobrevibacter* (*M. gottschalkii* clade and *M. ruminantium* clade), *Methanosphaera* (*M*. sp. A4) and Group 11 (*Candidatus Methanomethylophilus alvus*) varied significantly as a function of diet (Poisson regression, *P* < 0.01; 0.01; 0.01; 0.01). Overall members from the genus *Methanobrevibacter* were more abundant in the rumen of M calves, whereas *Methanosphaera* sp. A4 and *Candidatus Methanomethylophilus alvus* were more abundant in MC calves (TukeyHSD, *P* < 0.05). In addition, we observed that relative abundance of *Candidatus Methanomethylophilus alvus* decreased significantly (*P* < 0.001) with age (TukeyHSD, *P* < 0.05). Although the average abundance of *Methanosphaera* sp. A4 and *Methanobrevibacter gottschalkii* increased and decreased, respectively, over the developmental stages of MC calves, these variations were not significantly ascribed to age (*P* = 0.685; 0.467) or interaction of diet^*^age (*P* = 0.601; 0.241) (Tables 1–**3**).

**Table 1:** Diet effects on the relative abundance of archaeal, bacterial and fungal genera in the rumen of the dairy calves.

**Taxa**	**Diet^1^**		***P*-value**	**FDR^2^**
	**M**	**MC**		
**ARCHAEA**				
*Mbb. gottschalkii* clade	49.089 ± 8.21^a^	26.471 ± 8.789^b^	<0.001	<0.001
*Mbb. ruminantium* clade	14.098 ± 3.98^a^	8.555 ± 3.349^b^	<0.001	<0.001
*Methanosphaera* sp. A4	20.332 ± 6.288^b^	51.949 ± 9.972^a^	<0.001	<0.001
*Ca. M. alvus*	13.211 ± 5.335^a^	7.730 ± 3.309^b^	<0.001	<0.001
**BACTERIA**				
*Acidaminococcus*	0.533 ± 0.316^b^	1.130 ± 0.695^a^	<0.001	<0.001
*Bacteroides*	10.015 ± 3.346^a^	1.369 ± 0.645^b^	<0.001	<0.001
Bifidobacterium^**^	0.239 ± 0.153^b^	2.828 ± 1.352^a^	<0.001	<0.001
*Blautia*	1.026 ± 0.488^a^	0.478 ± 0.242^b^	0.031	0.050
Bulleidia^**^	0.303 ± 0.162^b^	2.161 ± 0.434^a^	<0.001	0.001
*Campylobacter*	0.226 ± 0.115^a^	0.146 ± 0.059^b^	<0.001	<0.001
CF231	0.439 ± 0.267^a^	0.040 ± 0.020^b^	<0.001	<0.001
*Corynebacterium*	0.935 ± 0.590^a^	0.227 ± 0.166^b^	<0.001	<0.001
Eubacterium^**^	0.011 ± 0.108^b^	0.098 ± 0.044^a^	<0.001	<0.001
Faecalibacterium^**^	0.043 ± 0.040^b^	0.207 ± 0.184^a^	<0.001	<0.001
*Lactobacillus*	3.351 ± 2.103^a^	0.364 ± 0.206^b^	<0.001	<0.001
*Megasphaera*	0.052 ± 0.026^b^	1.109 ± 0.482^a^	<0.001	<0.001
*Odoribacter*	0.361 ± 0.156^a^	0.036 ± 0.013^b^	<0.001	<0.001
*Oribacterium*	0.107 ± 0.091^a^	0.028 ± 0.017^b^	<0.001	<0.001
*p*-75-a5	0.392 ± 0.215^a^	0.020 ± 0.014^b^	<0.001	<0.001
*Parabacteroides*	1.208 ± 0.591^a^	0.175 ± 0.085^b^	<0.001	<0.001
Pseudoramibacter Eubacterium^**^	0.023 ± 0.007^b^	0.239 ± 0.076^a^	<0.001	<0.001
*Sharpea*	0.109 ± 0.050^b^	0.601 ± 0.263^a^	<0.001	<0.001
SHD231^**^	0.306 ± 0.211^a^	0.000 ± 0.000^b^	<0.001	<0.001
*Shuttleworthia*	1.346 ± 0.560^b^	5.100 ± 3.673^a^	<0.001	<0.001
*Streptococcus*	0.984 ± 0.516^a^	0.053 ± 0.021^b^	<0.001	<0.001
Succiniclasticum^**^	0.463 ± 0.253^b^	2.370 ± 0.644^a^	0.001	0.002
*Succinivibrio*	0.461 ± 0.312^b^	1.357 ± 0.765^a^	<0.001	<0.001
*Synergistes*	0.091 ± 0.079^a^	0.058 ± 0.022^a^	0.009	0.016
YRC22^**^	0.034 ± 0.019^b^	0.093 ± 0.039^a^	0.014	0.024
**FUNGI**				
*Caecomyces*	9.649 ± 2.770	19.197 ± 6.865	0.114	0.228
*Orpinomyces*	3.760 ± 2.296^a^	2.259 ± 0.854^a^	0.009	0.045
SK3	39.358 ± 5.882	38.278 ± 8.291	0.907	0.907
f_Neocallimastigaceae	37.339 ± 6.671	25.588 ± 7.399	0.245	0.327

*The values represent relative abundance, average percentage, and standard deviation*.

1 Calves fed with whole milk (M) or with whole milk plus starter concentrate ad libitum (MC);

2 P-value adjusted by FDR method; FDR < 0.05 were considered significant;

** *Genera that varied regardless of diet-age interaction*.

*Means between groups followed by the same letter are not significantly different (P > 0.05) by Tukey HSD test*.

### Bacterial populations vary with both age and diet

In our bacterial alpha-diversity analysis, Chao1 richness, inverse Simpson's diversity and Shannon's diversity did not vary significantly in response to diet (*P* = 0.490; 0.472; 0.496), age (ANOVA, *P* = 0.672; 0.490; 0.672) or the interaction of these factors (*P* = 0.490; 0.490; 0.496; Supplementary Table 5). Our beta-diversity analysis showed that Bray-Curtis dissimilarities in the bacterial community were attributed to diet and age (Permutation test, *P* = 0.001). The dissimilarities in the bacterial communities among M and MC calves persisted across age groups (28, 49, and 63 days old), as shown by sample clustering (Figure 1). In the M calves, dissimilarities in the bacterial communities decreased markedly with age, given the decrease of distance between samples of older calves (Figure 1B). However, the increase in similarity with age was less apparent in MC calves (Figure 1B). Our Venn diagram analysis showed that, out of 519 OTUs (at >0.1% relative abundance), only 37 and 42 of them were shared (by at least two calves in each group) across developmental stages (7, 28, 49, and 63 days) of both M and MC calves, respectively (Supplementary Figure 1). In both diet groups, the number of shared OTUs increased with age, indicating that bacterial community composition tended to be less heterogeneous among older calves (Supplementary Figure 1). Lastly, bacterial communities of M and MC calves had only 26 and 42 OTUs in common between age groups 7 and 63 days, and 28 and 63 days, respectively, thereby confirming the dissimilarities observed in the CAP analysis (Figure 1).

Our taxonomic composition analysis of the bacterial communities revealed a total of 1,125 OTUs (mean 112.5 ± SD 41.0) assigned to 20 phyla, 105 families or 140 genera. A total of 12 phyla, 41 families and 37 genera had relative abundances ≥0.1% and regardless of diet and age group, bacterial communities were dominated by the phyla Firmicutes (57.8 ± 3.4%), Bacteroidetes (28.2 ± 2.8%), Actinobacteria (5.2 ± 1.8%), Proteobacteria (3.9 ± 0.8%), Verrucomicrobia (1.7 ± 1.3%), and Synergistetes (1.3 ± 0.6%). Abundant families included the Ruminococcaceae (15.2 ± 1.7%), Lachnospiraceae (15.2 ± 2.2%), Prevotellaceae (11.7 ± 2.3%), Bacteroidaceae (6.2 ± 2.0%), Veillonellaceae (3.7 ± 0.8%), and Coriobacteriaceae (2.9 ± 1.6%). Lastly, the genera *Prevotella* (12.9 ± 2.3%), *Ruminococcus* (6.4 ± 1.1%), *Bacteroides* (6.2 ± 2.0%), *Shuttleworthia* (3.0 ± 1.7%), *Lactobacillus* (2.0 ± 1.2%), and *Butyrivibrio* (2.0 ± 0.5%) were more abundant and identified in at least 50% of all samples (Supplementary Figure 2; Figure 2).

Our analysis of deviance showed that the relative abundance of several bacterial genera varied according to diet (25 genera), age (27 genera), and the interaction of these factors (18 genera) (Tables 1–**3**). The bacterial genera that were not significantly different between diet, age or interaction are presented in Supplementary Table 6. A total of 8 bacterial genera varied significantly and exclusively according to diet group. The genera *Bifidobacterium, Bulleidia*, and *Succiniclasticum* (Poisson, *P* < 0.001; 0.001; 0.002, respectively) were more abundant (Tukey HSD, *P* < 0.05) in rumen samples of MC calves than in M calves (Table 1). Further, the relative abundance of 9 genera varied significantly and exclusively according to age group. The abundance of the genera *Pseudoramibacter, Eubacterium* and SHD-231 (Poisson, *P* < 0.001) increased significantly with age (TukeyHSD, *P* < 0.05) but did not reach at least 1% abundance in rumen samples from calves at 63 days old. In contrast, the genera *Bifidobacterium, Butyricimonas*, and *Oscillospira* (Poisson, *P* < 0.001; 0.028; 0.001) displayed abundances ≥0.5% in the rumen of calves at 7 days of age, but their proportion decreased (Tukey HSD, *P* < 0.05) in calves by 63 days old (Table 2).

**Table 2:** Age effect on the relative abundance of archaeal, bacterial, and fungal genera in the rumen of dairy calves.

**Taxa**	**Age**				***P*-value**	**FDR^1^**
	**7d**	**28d**	**49d**	**63d**		
**ARCHAEA**						
*Mbb. gottschalkii* clade	21.359 ± 15.109	46.633 ± 11.844	39.245 ± 11.53	40.018 ± 12.19	0.350	0.467
*Mbb. ruminantium* clade	0.647 ± 0.647	12.631 ± 3.702	13.585 ± 6.169	13.693 ± 5.114	0.075	0.150
*Methanosphaera* sp.A4	33.325 ± 17.933	28.982 ± 10.905	36.931 ± 11.729	37.13 ± 11.625	0.685	0.685
*Ca. M. alvus*	43.050 ± 17.134^a^	6.815 ± 2.814^b^	6.287 ± 4.132^b^	4.207 ± 2.575^b^	0.004	0.009
**Bacteria**						
*Acidaminococcus*	0.354 ± 0.322^b^	0.937 ± 0.636^a^	1.183 ± 0.932^a^	0.399 ± 0.151^b^	<0.001	<0.001
*Bacteroides*	12.281 ± 4.185^a^	11.681 ± 6.545^b^	3.138 ± 1.355^c^	1.403 ± 1.039^d^	<0.001	<0.001
Bifidobacterium^**^	0.745 ± 0.574^b^	4.245 ± 2.152^a^	0.388 ± 0.347^bc^	0.108 ± 0.093^c^	<0.001	<0.001
*Blautia*	2.930 ± 1.858^a^	0.257 ± 0.076^c^	0.673 ± 0.325^b^	0.372 ± 0.255^c^	<0.001	<0.001
Butyricimonas^**^	0.508 ± 0.233^a^	0.146 ± 0.096^b^	0.080 ± 0.067^b^	0.044 ± 0.036^b^	0.017	0.028
*Campylobacter*	0.006 ± 0.006^c^	0.341 ± 0.236^a^	0.175 ± 0.069^b^	0.152 ± 0.058^bc^	<0.001	<0.001
CF231	0.039 ± 0.028^b^	0.042 ± 0.025^b^	0.205 ± 0.105^b^	0.663 ± 0.546^a^	<0.001	<0.001
*Corynebacterium*	3.576 ± 2.245^a^	0.223 ± 0.148^b^	0.029 ± 0.018^b^	0.278 ± 0.278^b^	<0.001	<0.001
Eubacterium^**^	0.485 ± 0.445^a^	0.030 ± 0.017^b^	0.069 ± 0.057^b^	0.064 ± 0.025^b^	<0.001	<0.001
Faecalibacterium^**^	0.000 ± 0.000^b^	0.017 ± 0.006^b^	0.321 ± 0.251^a^	0.017 ± 0.014^b^	<0.001	<0.001
*Lactobacillus*	0.631 ± 0.305^c^	2.377 ± 1.853^b^	3.712 ± 3.249^a^	0.256 ± 0.179^c^	<0.001	<0.001
*Megasphaera*	0.039 ± 0.025^c^	1.309 ± 0.781^a^	0.248 ± 0.172^bc^	0.318 ± 0.175^b^	<0.001	<0.001
*Odoribacter*	0.259 ± 0.149^b^	0.455 ± 0.303^a^	0.161 ± 0.093^bc^	0.028 ± 0.013^c^	<0.001	<0.001
*Oribacterium*	0.000 ± 0.000^b^	0.008 ± 0.006^b^	0.058 ± 0.025^b^	0.189 ± 0.189^a^	<0.001	<0.001
Oscillospira^**^	2.009 ± 0.822^a^	0.484 ± 0.109^b^	0.242 ± 0.095^c^	0.452 ± 0.216^b^	0.001	0.001
p-75-a5	0.061 ± 0.054^c^	0.474 ± 0.329^a^	0.272 ± 0.253^b^	0.006 ± 0.006^c^	<0.001	<0.001
*Parabacteroides*	2.670 ± 2.344^a^	0.698 ± 0.407^b^	0.470 ± 0.193^b^	0.190 ± 0.140^c^	<0.001	<0.001
Phascolarctobacterium^**^	0.503 ± 0.218^a^	0.039 ± 0.013^b^	0.060 ± 0.032^b^	0.085 ± 0.085^b^	0.014	0.024
*Porphyromonas*	3.274 ± 1.271^a^	0.655 ± 0.515^b^	0.020 ± 0.012^c^	0.885 ± 0.882^b^	0.001	0.002
Pseudoramibacter^**^	0.022 ± 0.011^b^	0.050 ± 0.018^b^	0.166 ± 0.091^a^	0.177 ± 0.076^a^	<0.001	<0.001
*Sharpea*	0.006 ± 0.006^c^	0.572 ± 0.383^a^	0.251 ± 0.160^b^	0.340 ± 0.178^b^	<0.001	<0.001
SHD.231^**^	0.000 ± 0.000^b^	0.000 ± 0.000^b^	0.302 ± 0.293^a^	0.261 ± 0.258^a^	<0.001	<0.001
*Shuttleworthia*	1.905 ± 1.905^b^	0.549 ± 0.383^d^	6.916 ± 4.859^a^	1.157 ± 0.497^c^	<0.001	<0.001
*Streptococcus*	3.208 ± 1.943^a^	0.111 ± 0.032^b^	0.276 ± 0.186^b^	0.077 ± 0.033^b^	<0.001	<0.001
*Succinivibrio*	0.006 ± 0.006^c^	2.094 ± 1.326^a^	0.450 ± 0.266^b^	0.563 ± 0.351^b^	<0.001	<0.001
*Synergistes*	0.039 ± 0.028^b^	0.214 ± 0.163^a^	0.018 ± 0.011^b^	0.030 ± 0.019^b^	0.025	0.041
Treponema^**^	0.000 ± 0.000^b^	0.473 ± 0.237^a^	0.055 ± 0.032^b^	0.119 ± 0.083^b^	<0.001	<0.001
**FUNGI**						
*Caecomyces*	6.272 ± 3.753	9.499 ± 3.357	19.966 ± 8.191	14.211 ± 6.797	0.576	0.628
*Orpinomyces*	0.187 ± 0.187^b^	6.414 ± 4.625^a^	2.398 ± 1.054^b^	2.016 ± 1.222^b^	<0.001	<0.001
SK3	57.678 ± 8.36	24.587 ± 8.858	43.304 ± 9.3	38.532 ± 9.185	0.240	0.327
f_Neocallimastigaceae	34.354 ± 7.836	43.685 ± 11.661	19.253 ± 7.368	35.789 ± 9.952	0.149	0.256

*The values represent relative abundance, average percentage, and standard deviation*.

1 P-value adjusted by FDR method; FDR < 0.05 were considered significant;

** *Genera that varied regardless of diet-age interaction*.

*Means between age groups followed by the same letter are not significantly different (P > 0.05) by Tukey HSD test*.

The relative abundance of 18 genera varied simultaneously with diet and age (Table 3). This included the *Bacteroides, Parabacteroides, Lactobacillus*, and *Streptococcus* (Poisson, *P* < 0.001), which displayed high abundance in younger M calves (7 and or 28 days old), but their proportion decreased markedly and significantly (Tukey HSD, *P* < 0.05) in older calves (63 days old). In contrast, the abundance of *Parabacteroides, Lactobacillus*, and *Streptococcus* remained low and unchanged across the developmental stages of MC calves, while the *Bacteroides* increased significantly in MC calves at 63 days of age (Table 3). In addition, the abundance of the genera *Megasphaera, Sharpea*, and *Succinivibrio* increased significantly (Poisson, *P* < 0.001; Tukey HSD, *P* < 0.05) in the rumen of MC calves at 28 days (20 days after starter concentrate intake began) and decreased proportionally as age increased. Finally, the abundance of *Megasphaera* and *Sharpea* were significantly lower (TukeyHSD, *P* < 0.05) across the developmental stages of M calves, compared with MC calves (Table 3).

**Table 3:** Diet-age interaction effect on the relative abundance of archaeal, bacterial, and fungal genera in the rumen of dairy calves.

**Taxa**	Diet^*^age^1^							***P*-value**	**FDR^2^**
	**M_07d**	**M_28d**	**M_49d**	**M_63d**	**MC_28d**	**MC_49d**	**MC_63d**		
**ARCHAEA**									
*Mbb. gottschalkii* clade	21.359 ± 15.109	52.427 ± 16.81	59.977 ± 16.77	60.777 ± 15.239	40.839 ± 17.921	21.105 ± 13.666	19.259 ± 15.787	0.500	0.601
*Mbb. ruminantium* clade	0.647 ± 0.647	11.165 ± 3.962	25.494 ± 11.92	17.184 ± 5.623	14.097 ± 6.613	3.164 ± 1.692	10.202 ± 8.865	0.137	0.235
*Methanosphaera* sp.A4	33.325 ± 17.933	29.612 ± 14.107	10.345 ± 8.159	9.709 ± 8.955	28.351 ± 18.002	60.194 ± 17.33	64.552 ± 14.619	0.163	0.245
*Ca. M. alvus*	43.05 ± 17.134	4.531 ± 4.155	2.238 ± 1.493	4.854 ± 4.477	9.1 ± 3.936	9.83 ± 7.65	3.56 ± 2.994	0.639	0.685
**BACTERIA**									
*Acidaminococcus*	0.354 ± 0.322^c^	1.286 ± 1.286^b^	0.096 ± 0.069^c^	0.467 ± 0.257^c^	0.588 ± 0.281^c^	2.135 ± 1.726^a^	0.332 ± 0.181^c^	<0.001	<0.001
*Bacteroides*	12.281 ± 4.185^b^	22.652 ± 11.842^a^	5.302 ± 2.709^c^	0.611 ± 0.334^e^	0.710 ± 0.358^e^	1.244 ± 0.528^e^	2.194 ± 2.095^d^	<0.001	<0.001
*Blautia*	2.930 ± 1.858^a^	0.409 ± 0.110^bc^	0.718 ± 0.460^b^	0.100 ± 0.043^c^	0.105 ± 0.062^c^	0.634 ± 0.487^b^	0.644 ± 0.506^b^	<0.001	<0.001
*Campylobacter*	0.006 ± 0.006^b^	0.610 ± 0.464^a^	0.176 ± 0.055^b^	0.122 ± 0.038^b^	0.072 ± 0.026^b^	0.174 ± 0.124^b^	0.182 ± 0.115^b^	<0.001	<0.001
CF231	0.039 ± 0.028^c^	0.017 ± 0.011^c^	0.396 ± 0.207^b^	1.310 ± 1.071^a^	0.067 ± 0.050^c^	0.037 ± 0.033^c^	0.017 ± 0.011^c^	<0.001	<0.001
*Corynebacterium*	3.576 ± 2.245^a^	0.308 ± 0.294^bc^	0.010 ± 0.010^c^	0.000 ± 0.000^c^	0.139 ± 0.084^bc^	0.046 ± 0.033^c^	0.555 ± 0.555^b^	<0.001	<0.001
*Lactobacillus*	0.631 ± 0.305^c^	4.709 ± 3.595^b^	7.301 ± 6.963^a^	0.106 ± 0.034^c^	0.044 ± 0.038^c^	0.572 ± 0.448^c^	0.407 ± 0.361^c^	<0.001	<0.001
*Megasphaera*	0.039 ± 0.025^d^	0.011 ± 0.011^d^	0.048 ± 0.032^d^	0.110 ± 0.104^cd^	2.606 ± 1.418^a^	0.424 ± 0.317^bc^	0.525 ± 0.327^b^	<0.001	<0.001
*Odoribacter*	0.259 ± 0.149^b^	0.871 ± 0.579^a^	0.297 ± 0.193^b^	0.028 ± 0.016^c^	0.039 ± 0.026^c^	0.041 ± 0.024^c^	0.027 ± 0.022^c^	0.001	0.002
*Oribacterium*	0.000 ± 0.000^b^	0.006 ± 0.006^b^	0.053 ± 0.031^b^	0.378 ± 0.378^a^	0.011 ± 0.011^b^	0.062 ± 0.041^b^	0.000 ± 0.000^b^	<0.001	<0.001
p-75-a5	0.061 ± 0.054^c^	0.892 ± 0.637^a^	0.574 ± 0.540^b^	0.011 ± 0.011^c^	0.056 ± 0.044^c^	0.008 ± 0.008^c^	0.000 ± 0.000^c^	0.007	0.013
*Parabacteroides*	2.670 ± 2.344^a^	1.280 ± 0.768^b^	0.878 ± 0.364^b^	0.061 ± 0.023^c^	0.116 ± 0.067^c^	0.112 ± 0.036^c^	0.319 ± 0.281^c^	<0.001	<0.001
*Porphyromonas*	3.274 ± 1.271^a^	1.304 ± 0.999^c^	0.019 ± 0.010^d^	0.006 ± 0.006^d^	0.006 ± 0.006^d^	0.021 ± 0.021^d^	1.765 ± 1.765^b^	0.014	0.024
*Sharpea*	0.006 ± 0.006^d^	0.116 ± 0.069^cd^	0.182 ± 0.137^cd^	0.121 ± 0.121^cd^	1.027 ± 0.748^a^	0.312 ± 0.284^bc^	0.559 ± 0.325^b^	<0.001	<0.001
*Shuttleworthia*	1.905 ± 1.905^b^	0.954 ± 0.748^bc^	1.427 ± 1.042^b^	1.084 ± 0.670^bc^	0.145 ± 0.138^c^	11.719 ± 8.982^a^	1.230 ± 0.797^b^	<0.001	<0.001
*Streptococcus*	3.208 ± 1.943^a^	0.149 ± 0.056^c^	0.578 ± 0.380^b^	0.067 ± 0.029^c^	0.072 ± 0.028^c^	0.012 ± 0.009^c^	0.088 ± 0.064^c^	<0.001	<0.001
*Succinivibrio*	0.006 ± 0.006^d^	1.131 ± 1.124^b^	0.010 ± 0.006^d^	0.772 ± 0.694^bc^	3.058 ± 2.470^a^	0.835 ± 0.469^bc^	0.354 ± 0.208^cd^	<0.001	<0.001
*Synergistes*	0.039 ± 0.028^b^	0.335 ± 0.328^a^	0.005 ± 0.005^b^	0.000 ± 0.000^b^	0.094 ± 0.061^b^	0.029 ± 0.019^b^	0.061 ± 0.036^b^	<0.001	<0.001
**FUNGI**									
*Caecomyces*	6.272 ± 3.753	10.189 ± 5.71	8.01 ± 3.485	13.924 ± 8.662	8.924 ± 4.41	30.427 ± 14.457	14.498 ± 11.324	0.418	0.502
*Orpinomyces*	0.187 ± 0.187^b^	10.566 ± 10.332^a^	2.293 ± 0.988^b^	2.778 ± 2.415^b^	2.954 ± 1.417^b^	2.491 ± 1.853^b^	1.254 ± 0.71^b^	0.015	0.045
SK3	57.678 ± 8.36	10.379 ± 6.15	39.979 ± 11.37	47.517 ± 11.01	36.427 ± 14.17	46.214 ± 14.991	29.548 ± 14.754	0.046	0.111
f_Neocallimastigaceae	34.354 ± 7.836^c^	61.119 ± 17.932^a^	35.201 ± 13.59^c^	22.503 ± 9.784^d^	29.156 ± 13.832^cd^	5.298 ± 2.313^e^	49.074 ± 16.415^b^	0.015	0.045

*The values represent relative abundance, average percentage, and standard deviation*.

1 Calves fed with only whole milk (M) or whole milk and starter concentrate (MC) that were slaughtered at 7, 28, 49, and 63 days old;

2 P-value adjusted by FDR method;

*FDR < 0.05 were considered significant; Means between groups followed by the same letter are not significantly different (P > 0.05) by Tukey HSD test*.

### Fungal populations vary with both age and diet

In our fungal alpha-diversity analysis, Chao1 richness, inverse Simpson's diversity and Shannon's diversity did not differ by diet (ANOVA, *P* = 0.967; 0.967; 0.967), age (*P* = 0.967; 0.967; 0.967) or the interaction of these factors (*P* = 0.967; 0.967; 0.385; Supplementary Table 5). Our beta-diversity analysis showed that Bray-Curtis dissimilarities in the fungal community were significantly ascribed to diet^*^age (Permutation test, *P* = 0.014), whereas variations observed with diet (*P* = 0.832) and age (*P* = 0.405) were not significant (Figure 1). The dissimilarities in the fungal communities were more evident among M and MC calves at 49 days old, where diet groups tended to cluster separately (Figure 1). However, this segregation was not observed among calves at 63 days old where community composition was more homogeneous. In addition, our Venn diagram analysis showed that, out of 48 OTUs (at ≥0.1% relative abundance in at least one sample), only 3 and 4 of them were shared across all developmental stages of M and MC calves, respectively (Supplementary Figure 1).

Our taxonomic composition analysis of the fungal communities revealed a total of 84 OTUs (mean 6.9 ± SD 2.5) assigned to the family Neocallimastigaceae (99.98 ± 0.02%) including the genera SK3 (38.9 ± 4.9%), *Caecomyces* (14.1 ± 3.6%) and *Orpinomyces* (3.1 ± 1.3%) that were identified in at least 50% of all samples, while the genera *Piromyces* (4.4 ± 2.4%), *Neocallimastix* (3.9% ± 2.1%), AL4 (1.6 ± 1.0), AL5 (1.6 ± 1.2), *Anaeromyces* (0.3 ± 0.1%), AL8 (0.1 ± 0.0%), and DTI (0.1 ± 0.1%) were observed in <40% of all samples. Only 25.6% of sequences were assigned to the species level including *Caecomyces* 1 (14.1 ± 3.6%), that was identified in 60% of all samples while *Neocallimastix* 1 (3.9 ± 2.1%), Piromyces 7 (3.4 ± 2.3%), *Orpinomyces* 4 (2.8 ± 1.3%), *Piromyces* 4 (1.0 ± 1.0%), and *Anaeromyces* 1 (0.3 ± 0.1%) were identified in <15 calves out of 43. The distributions of fungal taxa among individual calves and diet-age groups are displayed in Supplementary Figure 2 and Figure 2, respectively.

Our analysis of deviance showed that the relative abundances of the genus *Orpinomyces* and the family Neocallimastigaceae varied simultaneously with diet and age (Poisson regression, *P* = 0.045; 0.045). In the M calves, the abundance of *Orpinomyces* and the family Neocallimastigaceae increased significantly at 28 days of age but decreased in older calves. In contrast, the abundance of *Orpinomyces* did not change across developmental stages of MC calves, while the Neocallimastigaceae decreased markedly at 49 days, but increased in calves 63 days old. Conversely, variations observed in the abundance of the genera SK3 and *Caecomyces* were not significantly ascribed to diet (Poisson regression, *P* = 0.907; 0.228), age (*P* = 0.327; 0.628), or interaction (*P* = 0.111; 0.502) (Tables 1–3).

### Co-occurrence and correlation analysis

**Figure 3:**
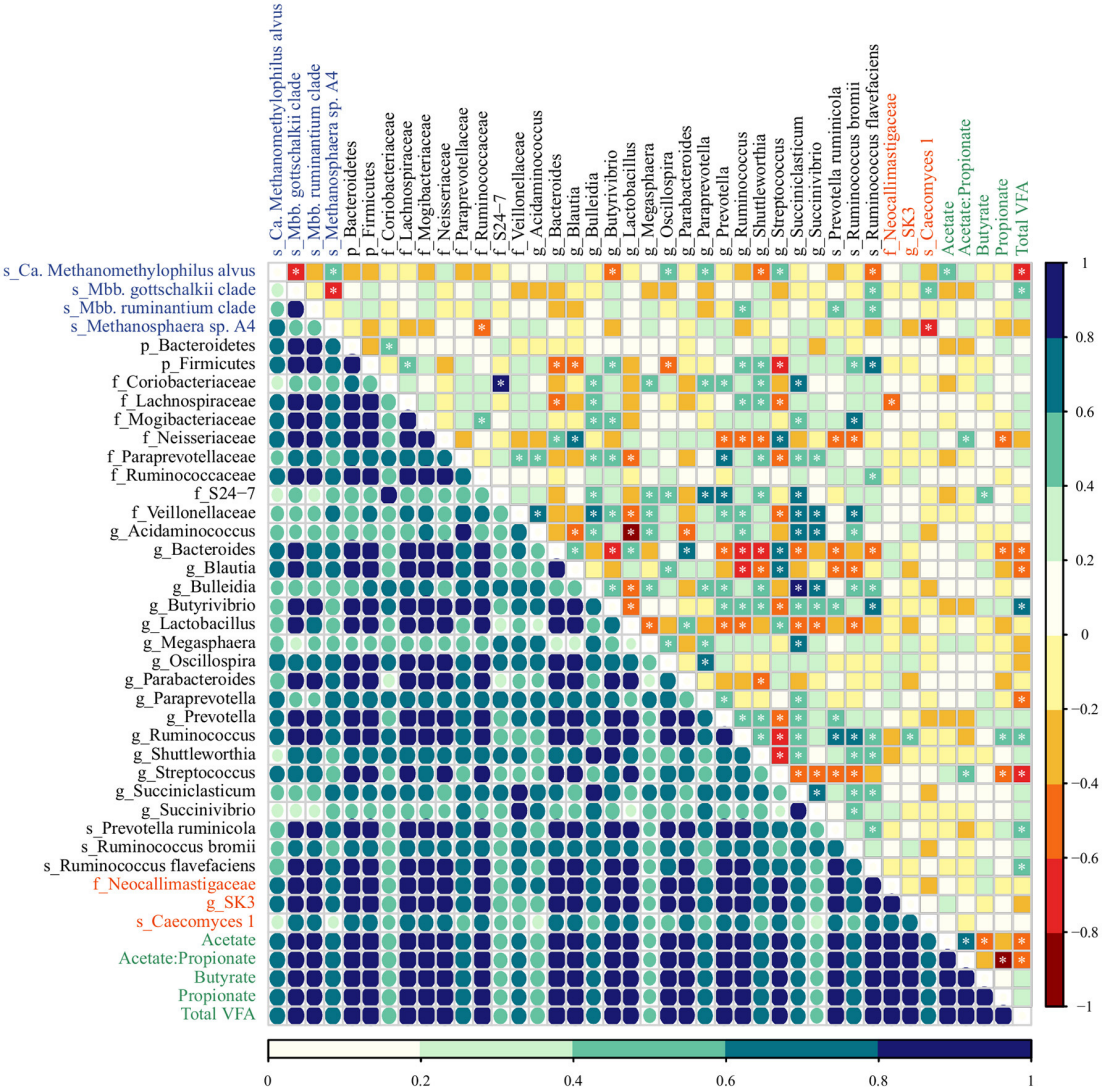
The co-occurrence and correlation among archaeal (blue), bacterial (black), fungal (red) taxa, and molar proportions of volatile fatty acids (green) in the rumen samples of M-fed calves. The co-occurrence is shown by Dice's index (lower matrix), ranging from 0 to 1, represented by color key (dark blue: high, seagreen: moderate, light blue: low co-occurrence). The correlation is shown by Spearman's rank correlation (upper matrix), ranging from −1 to 1 and represented by color key dark red (perfect negative correlation) to dark blue (perfect positive correlation). The correlations marked with asterisks are significant at a *P* < 0.05 (Supplementary Table 7).

**Figure 4:**
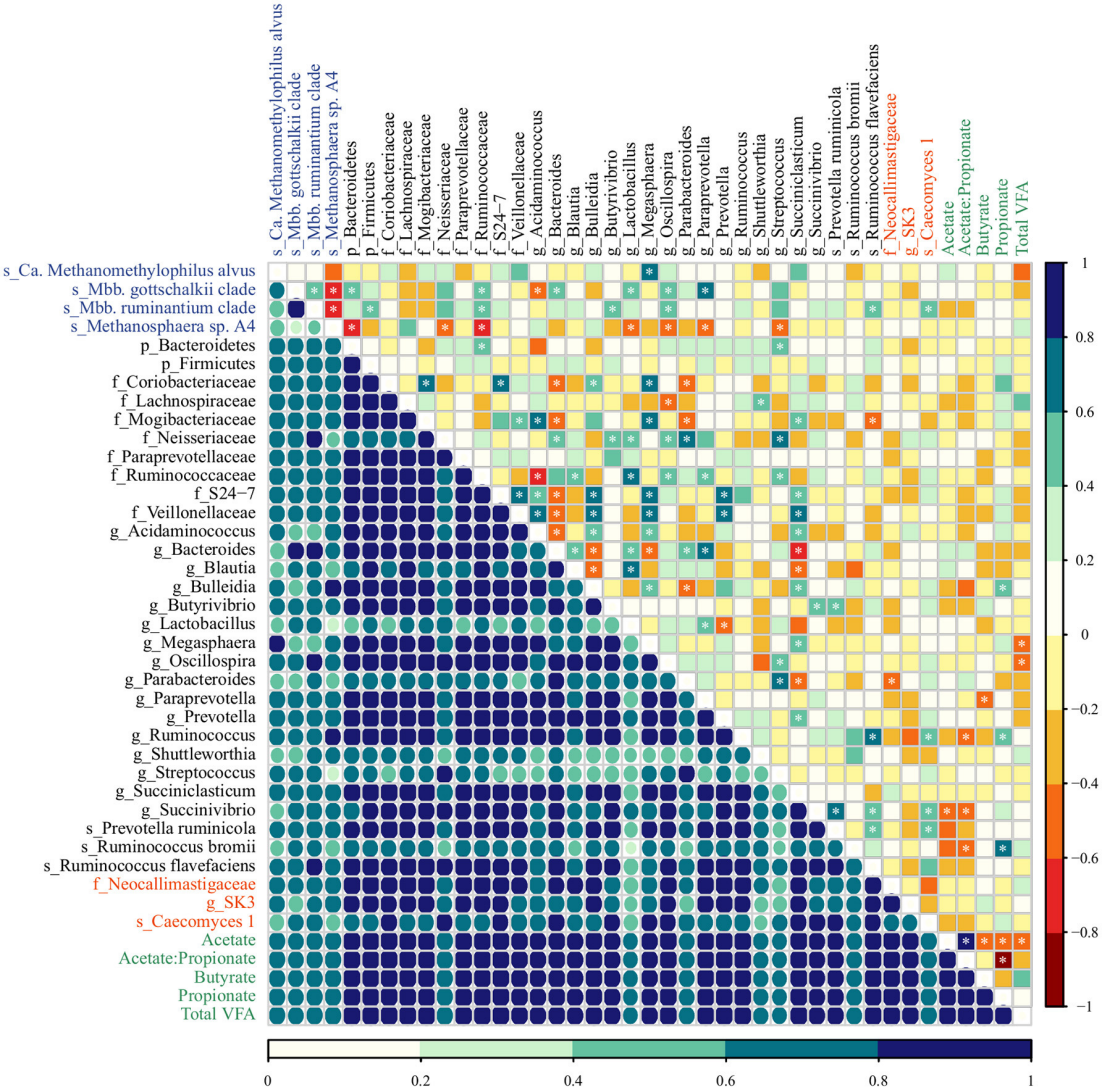
The co-occurrence and correlation among archaeal (blue), bacterial (black), fungal (red) taxa, and molar proportions of volatile fatty acids (green) in the rumen samples of MC-fed calves. The co-occurrence is shown by Dice's index (lower matrix), ranging from 0 to 1, represented by color key (dark blue: high, seagreen: moderate, light blue: low co-occurrence). The correlation is shown by Spearman's rank correlation (upper matrix), ranging from −1 to 1 and represented by color key dark red (perfect negative correlation) to dark blue (perfect positive correlation). The correlations marked with asterisks are significant at a *P* < 0.05 (Supplementary Table 7).

The co-occurrence and correlation among the most abundant taxa (4 archaea, 29 bacteria, and 3 fungi) and molar proportion of VFAs (acetate, propionate, butyrate, total VFA, and acetate-to-propionate ratio) in rumen samples were assessed in each diet group using two approaches: the Dice index which indicates no, to moderate, to high co-occurrence (ranges from 0 to 1) and Spearman's rank correlation, which indicates perfect negative to perfect positive correlation (ranges from −1 to 1). Several inter-intra microbial associations were observed in the rumen of pre-weaned calves but the type and extent of these associations varied in response to diet (Figures 3, 4; Supplementary Table 7).

In M calves, the main associations between bacterial taxa were the high co-occurrence and positive correlation of the genera *Succiniclasticum* and *Succinivibrio* (Dice = 0.80; Spearman = 0.66, *P* < 0.001), which in turn, were positively correlated with *Bulleidia* (Dice = 0.87, 0.74; Spearman = 0.88, 0.66, *P* < 0.001, < 0.001). In addition, we observed that members from the genus *Bacteroides* (abundant in the rumen of M calves, Table 1) displayed a high co-occurrence and positive correlation with *Streptococcus* (Dice = 0.81; Spearman 0.62, *P* = 0.001) and *Parabacteroides* (Dice = 0.88; Spearman 0.64 *P* = 0.001) and these taxa were negatively correlated with the genera *Ruminococcus* (Dice = 0.94, 0.78; Spearman = −0.76, −0.66, *P* < 0.001, < 0.001) and *Shuttleworthia* (Dice = 0.68, 0.50, 0.61; Spearman = −0.66, −0.71, −0.41, *P* < 0.001, < 0.001, 0.044). For the archaea, *Methanosphaera* sp. A4 was positively associated with *Ca. Methanomethylophilus alvus* (Dice = 0.74; Spearman = 0.52, *P* = 0.008) and both taxa were negatively correlated with *Mbb. gottschalkii* (Dice = 0.50, 0.39; Spearman = −0.70, −0.76, *P* < 0.001, < 0.001). *Methanosphaera* sp. A4 and *Ca. Methanomethylophilus alvus* also displayed negative association with bacterial members from Ruminococcaceae family (Dice = 0.72; Spearman = −0.54, *P* = 0.006) and the genus *Butyrivibrio* (Dice = 0.48, Spearman = −0.55; *P* = 0.005). In addition, we observed potential association among fungal taxa and methanogens: *Caecomyces* 1 displayed low co-occurrence and negative correlation with *Methanosphaera* sp. A4 (Dice = 0.30; Spearman = −0.61, *P* = 0.001) and positive correlation with *Mbb. gottschalkii* (Dice = 0.71; Spearman = 0.51, *P* = 0.009). Lastly, fungal and bacterial taxa displayed moderate co-occurrence (Dice = 0.69) but only weak associations(Spearman <0.5 or > −0.5) were observed (Figure 3; Supplementary Table 7).

In MC calves, the main associations between bacterial taxa included a negative correlation between *Bacteroides* and *Bulleidia* (Dice = 0.79; Spearman = −0.60, *P* = 0.005) as well as *Succiniclasticum* (Dice = 0.82; Spearman −0.64, *P* = 0.002). Similar to M calves, *Succiniclasticum* displayed positive association with *Bulleidia* and *Prevotella* (Dice = 0.91, 0.95; Spearman = 0.57, 0.59, *P* = 0.009, 0.006) while the genus *Succinivibrio* displayed high co-occurrence and positive correlation with *Prevotella ruminicola* (Dice = 0.83; Spearman = 0.69, *P* = 0.001). For associations between methanogens, *Mbb. gottschalkii* and *Mbb. ruminantium clade* displayed high co-occurrence and positive correlation (Dice = 0.96; Spearman = 0.53, *P* = 0.017) and both taxa were negatively correlated with *Methanosphaera* sp. A4 (Dice = 0.33, 0.40; Spearman = −0.76, −0.71, *P* < 0.001, < 0.001). *Methanosphaera* sp. A4 was negatively correlated with bacterial members from the phylum Bacteroidetes (Dice = 0.75; Spearman = −0.60; *P* = 0.005) and the family Ruminococcaceae (Dice = 0.79, Spearman = −0.62; *P* = 0.004). In addition, *Ca. Methanomethylophilus alvus* and *Mbb. gottschalkii* displayed a positive association with genera *Megasphaera* (Dice = 0.81; Spearman = 0.75, *P* < 0.001) and *Paraprevotella* (Dice = 0.73; Spearman = 0.78, *P* < 0.001) in MC calves. Lastly, we observed moderate association between fungal and bacterial taxa: *Caecomyces* 1 displayed high co-occurrence and moderate association with *Ruminococcus* (Dice = 0.77; Spearman = 0.57, *P* = 0.009) and *Succinivibrio* (Dice = 0.85; Spearman = 0.53, *P* = 0.017).

In regards to VFAs, the proportion molar of acetate, propionate and butyrate increased proportionally to the age of MC calves (Anova, *P* < 0.001; 0.035; 0.033) as a result of starter intake, while M calves displayed lowest VFA concentration as a result of solid restriction (Supplementary Table 8). Regardless of the diet group, we identified few strong associations between molar proportions of VFAs and microbial taxa (Figures 3, 4; Supplementary Table 7). In M calves, Total VFAs displayed positive association with *Butyrivibrio* (Dice = 0.89; Spearman = 0.69, *P* < 0.01) and negative correlation with *Ca. Methanomethylophilus alvus* (Dice = 0.68; Spearman = −0.68, *P* < 0.01) and *Streptococcus* (Dice = 0.84; Spearman = −0.61, *P* = 0.001). In MC calves, the molar proportion of acetate was negatively associated with *Succinivibrio* (Dice = 0.82; Spearman = −0.58, *P* = 0.007) while propionate was positively associated with *Ruminococcus bromii* (Dice = 0.71; Spearman = 0.70, *P* = 0.001) (Figures 3, 4; Supplementary Table 7).

## Discussion

Overall, the process by which microorganisms colonize the rumen of developing calves has not been fully elucidated. Studies performed with culture-based techniques suggested that microbial colonization is sequential because bacteria are the first microbial group found in the neonatal rumen (1–2 days old), followed by methanogenic archaea (calves 3–14 days of age), anaerobic fungi (lambs 8–10 days of age), and then protozoa (calves > 56 days of age) (Anderson et al., 1987a; Fonty et al., 1987; Minato et al., 1992). However, more recent work with molecular techniques identified archaea and bacteria in the rumen within hours of birth (Skillman et al., 2004; Gagen et al., 2012; Guzman et al., 2015), suggesting that initial colonization occurs prior to or during calving. Moreover, little is known regarding how important early-life factors like diet impact the colonization and persistence of microbes in the rumen. To address this, we characterized the archaeal, bacterial, and fungal communities in the rumen of pre-weaning dairy calves fed two diets (M: milk-fed and MC: milk plus starter concentrate fed) across four developmental stages (7, 28, 49, and 63 days old) and investigated the impact of diet on the composition and abundance of pre-weaning rumen communities.

The results of our study reveal that the main microbial groups commonly found in the mature rumen are established in the rumen of dairy calves at 1 week of age. These include methanogenic archaea, (*Mbb. gottschalkii* clade, *Methanosphaera* sp. A4 and *Ca. Methanomethylophilus alvus*), bacteria known to occupy different metabolic niches (*Bacteroides, Butyrivibrio, Lactobacillus, Megasphaera, Prevotella, Ruminococcus, Streptococcus*) and anaerobic fungi with known prominent roles in fiber degradation (*Caecomyces, Orpinomyces*, SK3). These results show that colonization of archaeal, bacterial, and fungal communities occur prior to the introduction of solid food (Bryant et al., 1958; Fonty et al., 1987; Rey et al., 2014; Guzman et al., 2016) and supports the suggestion that microbiota-related interventions could be viable right after birth (Abecia et al., 2013).

The main substrate for rumen microorganisms in calves prior to solid feed intake is liquid colostrum, milk or milk replacer that fails to flow directly to the abomasum through the esophageal groove and instead, enters the rumen (Wise and Anderson, 1939; Wise et al., 1942; Anderson et al., 1987b; Rey et al., 2012b). The bucket feeding system used in this study increases the frequency of esophageal groove failure and the amount of liquid food entering the rumen (Wise and Anderson, 1939; Wise et al., 1942; Labussière et al., 2014). Thus, it is not surprising that we identified VFAs in ruminal samples from 7-day-old calves fed exclusively colostrum and milk (Figure 3; Supplementary Table 8). These findings support the model that some bacteria and fungi establish in the rumen early on in life, and utilize milk nutrients such as lactose and carbon as energy sources (Russell and Baldwin, 1978; Marounek et al., 1988; Phillips and Gordon, 1995; Rainey, 2009). Further, the presence of members from the archaeal genus *Methanobrevibacter* in the rumen of M calves may be favored by lactose-fermenting bacteria and fungi that release hydrogen (H_2_) and carbon dioxide (CO_2_), both of which are substrates for methanogenesis (Bauchop and Mountfort, 1981; Wolin and Miller, 1983).

However, milk-associated nutrients fail to explain the presence of members from the genus *Methanosphaera* sp. A4 in very young calves. *Methanosphaera* sp. is known to obtain energy exclusively from the reduction of methanol. Methanol is produced in the rumen through the hydrolysis of pectin, a carbohydrate absent in milk (Miller and Wolin, 1985; Pol and Demeyer, 1988; Fricke et al., 2006). Therefore, we speculate that alternative pathways for methanol production may be present (Dorokhov et al., 2015), or that some *Methanosphaera* sp. capable of utilizing other substrates exist in the rumen of M calves. Likewise, the spillage of milk into the rumen may not be directly responsible for the presence of members from genus *Succiniclasticum*, which convert succinate to propionate as their sole energy-yielding mechanism (van Gylswyk, 1995). We speculate that establishment of *Succiniclasticum* may be supported by *Bulleidia* and *Succinivibrio* (succinate-producers), given the positive association among these genera in the rumen of M calves (Figure 3). This strong and positive association between *Succiniclasticum* and *Succinivibrio* in M calves was not observed in MC calves (Figure 4), likely due to the greater availability of succinate resulting from starch fermentation by other succinate-producers. Overall, our data suggests the existence of opportunistic associations between rumen microorganisms in response to diet.

While the establishment of the microbiota occurs regardless of solid food intake, diet was a strong determinant of the abundance of bacterial and archaeal taxa in the rumen of calves. In the bacterial community of MC calves, the introduction of starter concentrate intake promoted an increase in the relative abundance of taxa known to degrade readily fermentable carbohydrates (i.e., *Succinivibrio, Sharpea, Megasphaera*) at 28 days of age, although these taxa did not persist with increasing age. In contrast, we observed that the bacterial community in the rumen of M calves was dominated by taxa from the genera *Lactobacillus, Bacteroides*, and *Parabacteroides*, whose abundances decreased with age but remained higher than in MC calves (Table 3). This suggests that diet is an important factor that affects the bacterial community over this short time frame, potentially by acting as a selective mechanism for taxa adapted to new substrates. However, the persistence of diet-related responses in older calves likely depends on other factors that accompany increasing age, such as ruminal development and host-microbe interactions.

Consistent with our results, previous work has shown that the genus *Succinivibrio* is present in low proportions in the rumen of calves at 2 days old and that its abundance increases after the provision solid food (Rey et al., 2014), followed by a subsequent decrease post-weaning (Meale et al., 2016). The primary *Succinivibrio* found in the rumen (*S. dextrinosolvens*) ferments starch hydrolysis products (maltose, dextrin, glucose) into primarily succinate, acetate, and formate (Bryant and Small, 1956) and has a negative association with ruminal pH (Rey et al., 2014) and molar proportion of acetate (Figure 4).

Another specialist genus whose abundance increased in response to calf starter was *Megasphaera*. In a similar study, the abundance of this genus was very low in the rumens of neonate calves, and displayed a tendency to increase with age or solid food provision (Jami et al., 2013). In contrast, *Megasphaera* was identified at low proportion (0.4–0.6%) in the rumens of calves pre- and post-weaning (Meale et al., 2016), and its presence was not reported in studies performed with calves fed milk replacer (Li et al., 2012; Wu et al., 2012) or milk with starter concentrate and hay (Malmuthuge et al., 2014; Rey et al., 2014). Taken together, these results suggest that the abundance of *Megasphaera* in the developing rumen is low, and that dietary strategies to promote its colonization may be of interest, as members of this genus, like *M. elsdenii*, are known as efficient lactate-utilizing bacteria whose activities result in propionate and butyrate production, VFAs known to stimulate the development and differentiation of the rumen epithelium (Counotte et al., 1981; Malhi et al., 2013). Moreover, the activity of *M. elsdenii* may prevent the accumulation of lactic acid from lactate-producers, thereby minimizing ruminal acidosis in calves during adaptation to starter concentrate (Anderson et al., 1987b; Quigley et al., 1992).

In addition to the ruminal bacteria, we found that the relative abundance of methanogens was responsive to the inclusion of concentrate in the calf diet. Previous reports indicate that *Methanobrevibacter* are dominant in the rumens of calves fed milk plus starter concentrate (Rey et al., 2012a; Zhou et al., 2014), and that *Methanosphaera* increases markedly after the beginning of solid food intake (Rey et al., 2012a). Here, we observed higher relative abundances of *Methanosphaera* sp. A4 in the rumens of MC calves. This is likely directly attributed to diet composition, as pectin was present in the main ingredients (Malathi and Devegowda, 2001) of the starter concentrate used in our study (Supplementary Table 1). Thus, we posit that the methanol produced through the fermentation of pectin is made available to *Methanosphaera* sp. A4 as well as methanol-utilizing bacteria such as *Eubacterium*, whose abundance was also significantly higher in the rumen of MC calves (Table 1). Although *Ca. Methanomethylophilus alvus* would be able to reduce methanol and or other methylated compounds for methanogenesis (Borrel et al., 2012), the diet composition did not promote its colonization in the rumen of MC calves (Table 1). Indeed, in both diet groups the abundance of *Ca. Methanomethylophilus alvus* decreased markedly with age (Tables 2, 3), probably due to competition with other methanogens (Figure 3).

Bacterial changes resulting from the inclusion of concentrate intake (i.e., increase in the abundance of amylolytic populations) can also lead to decreases in the acetate-to-propionate ratio and H_2_ availability within the rumen (Johnson and Johnson, 1995; Martin et al., 2010), which in turn affects the archaeal community (Table 1). These dynamics may reflect changes in the archaeal community by favoring *Methanosphaera* sp. that require only 1 mol of H_2_ to produce 1 mol of CH_4_, as opposed to *Methanobrevibacter* species that require 4 mols of H_2_ in order to produce 1 mol of CH_4_ (Miller and Wolin, 1985; Fricke et al., 2006; Sun et al., 2015). Moreover, the abundance of *Methanobrevibacter* in our study was negatively correlated with *Methanosphaera* across developmental stages of M and MC calves (Figures 3, 4), possibly due to competition for H_2_ within the rumen (Miller and Wolin, 1985; Janssen and Kirs, 2008).

In contrast to the ruminal archaea and bacteria, diet and age did not affect the rumen fungal communities in our study. Members from the genera *Caecomyces* and SK3 were identified in at least 60% of all calves at 7 days old, remained abundant, and were irresponsive to the inclusion of starter concentrate. In addition, we observed that members from the genera *Anaeromyces, Piromyces, Neocallimastix*, and DT1 did not establish in all calves and were not identified as part of the core fungal community in M or MC calves. These results are comparable to calves raised on milk plus starter pellets, where rumen fungi were highly variable pre-weaning and not dramatically impacted by diet (milk-replacer plus calf starter) until the weaning transition (Dill-McFarland et al., 2017). In contrast, Fonty et al. (1987) identified *Neocallimastix frontalis* and *Caecomyces communis* (previously *Sphaeromonas communis sensu Orpin*) in the rumen of lambs at 8–10 days old, although these fungi disappeared in almost all of the lambs after concentrate and hay were offered. Given the importance of rumen fungi in fiber degradation, these results may indicate that fungi do not effectively establish until a continual influx of fiber is available to the rumen, although further work is needed to confirm this hypothesis.

The fungal genera identified in our study (*Neocallimastix, Orpinomyces*, and *Piromyces*) and in previous reports (Fonty et al., 1987; Dill-McFarland et al., 2017) include species well known for fiber degradation; many of these species are also able to utilize starch and its breakdown products. The extent to which this occurs depends on the fungal species and carbohydrate source (Phillips and Gordon, 1988, McAllister et al., 1993). Starch fermentation is a sought-after niche in the rumen, and the slow growth rate of fungi, relative to bacteria, constitutes a limiting factor for competition of these substrates (McAllister et al., 1993). The rapid release of acetate, formate and lactate from starch fermentation causes a drop in ruminal pH that may inhibit fungal growth (Srinivasan et al., 2001). Collectively these factors have been ascribed to decreases in the rumen fungal community of adult ruminants fed a grain-rich diet, relative to animals on a forage-based diet (Denman et al., 2008; Boots et al., 2013; Kumar et al., 2015).

In summary, our results provide new insights into the colonization and associations within the microbiota of the developing calf rumen. We found that archaeal, bacterial and fungal communities co-occur in the rumen early on during calf development but are impacted differently by pre-weaning diet and age. We observed that the inclusion of starter concentrate significantly affected rumen bacterial communities by promoting increases in bacteria known to readily degrade fermentable carbohydrates and depressing those reliant on milk components like lactose. These bacterial changes likely resulted in apparent diet-driven differences in the archaeal community, likely due to altered fermentation patterns and availability of hydrogen in the rumen. No such differences were found for ruminal fungi, likely due to the low availability of fiber content in the provided concentrate. These results indicate that pre-weaning manipulation of the rumen microbiota may be possible through dietary intervention and our study serves as a useful framework for designing strategies aimed at promoting colonization of target microbes linked to improved development of the calf.

## Author contributions

JD, MM, and FM designed the experiment. FM, HM, and GS provided experimental and laboratorial resources. JD conducted the research, sample collection and DNA extraction. JD and KD performed library construction and sequencing. JD, KD, MN, and RR conducted data analyses and interpretation of results. JD wrote the manuscript. MM, KD, FM, HM, and GS reviewed and edited the manuscript. All authors read and approved the final manuscript.

### Conflict of interest statement

The authors declare that the research was conducted in the absence of any commercial or financial relationships that could be construed as a potential conflict of interest.
