# Supplementary material for: Effect of Pre-weaning Diet on the Ruminal Archaeal, Bacterial, and Fungal Communities of Dairy Calves
Source: Front Microbiol. 2017 Aug 15;8:1553. doi: 10.3389/fmicb.2017.01553 (PMC5559706; doi:10.3389/fmicb.2017.01553)
Supplement: Supplementary file 1 [file DataSheet1.PDF]

Supplementary Material

Effect of pre-weaning diet on the ruminal archaeal, bacterial and fungal communities of dairy calves

Juliana Dias[1,2], Marcos Inácio Marcondes[1], Melline Fontes Noronha[3], Rafael Tassinari Resende[4], Fernanda Samarini Machado[5], Hilário Cuquetto Mantovani[6], Kimberly A. Dill-McFarland[7], Garret Suen[7**]**

[1]Department of Animal Science, Universidade Federal de Viçosa, Viçosa, MG, Brazil, [2]Coordenação de Aperfeiçoamento de Pessoal de Nível Superior, Ministério da Educação, Brasília, Brazil, [3]Division of Microbial Resources, Research Centre for Chemistry, Biology and Agriculture, University of Campinas, Campinas, SP, Brazil, [4]Department of Statistics, Universidade Federal de Viçosa, Viçosa, MG, Brazil, [5]Embrapa Dairy Cattle, Juiz de Fora, MG, Brazil, [6]Department of Biology, Universidade Federal de Viçosa, Viçosa, MG, Brazil, [7]Department of Bacteriology, University of Wisconsin-Madison, Madison, WI, USA

***Corresponding author:**

Kimberly A. Dill-McFarland, dillmcfarlan@wisc.edu
Garret Suen, gsuen@wisc.edu

1 Supplementary Figures and Tables

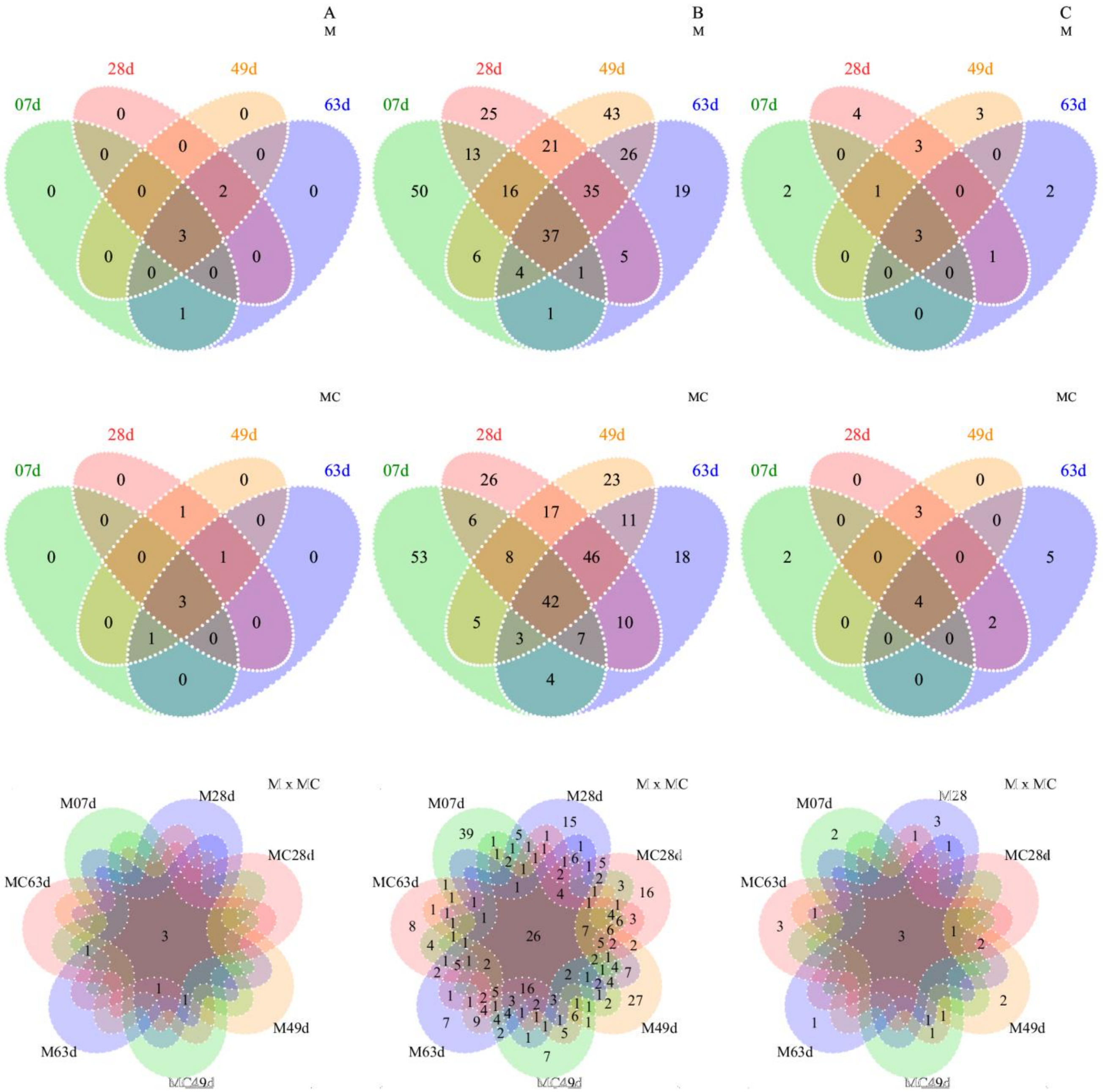

Supplementary Figure 1. Venn diagrams of shared archaeal (A), bacterial (B), and fungal (C) OTUs among calves according to diet (M: milk-fed or MC: milk and starter concentrate fed) and age group (7, 28, 49 and 63 days old). In all plots of MC calves, the M-07d group was included as a reference. Only OTUs at > 0.1% relative abundance and present in at least two rumen samples in each diet-age groups were included.

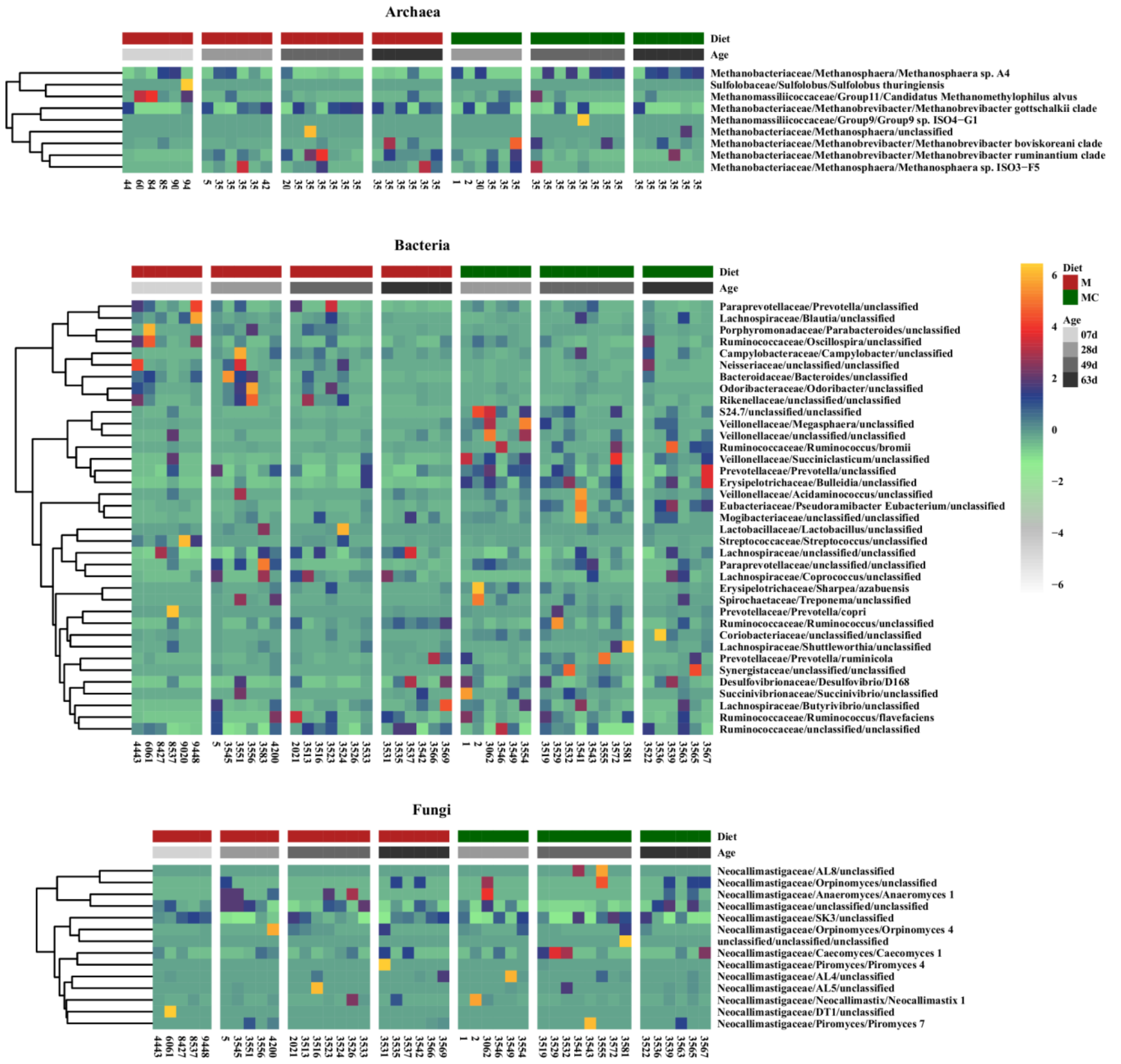

Supplementary Figure 2. Distribution of the archaeal, bacterial and fungal taxa (OTUs summarized at family/genus/specie) among individual calves grouped according to diet (M: milk-fed and MC: milk and starter concentrate fed) and age (7, 28, 49 and 63 days old). Only bacterial taxa at abundance ≥0.5% in at least one sample and present in at least 50% of all samples are show. Colors in the horizontal bars at the top of the plot represent diet (M: dark red, MC: dark green) and age (7 days: light grey, 28 days: grey medium, 49 days: grey medium-dark and 63 days old: dark grey). The scale (Z-score) represents the relative abundance at gradient of color from light grey (low abundance) to gold (high abundance). The hierarchical dendrogram was established through Minkowski distances of the taxa along the y axis and Ward.D2 linkage clustering method.

Table S1. Chemical composition of whole milk and starter concentrate fed to calves during the experimental period (7 to 63 days).

Composition	Milk	Starter concentrate[1*]
Dry matter (%)	12.32	89.33
Crude protein (DM basis %)	25.89	17.45
Ether extract (DM basis %)	37.64	1.940
Neutral Detergent Fiber (DM basis %)	-	12.99
Non-Fiber Carbohydrate (DM basis %)	31.23	62.62
Ashes (DM basis %)	5.240	5.01

[1]Contained 62.637% corn ground, 32.486% soybean meal, 3.094% wheat meal, 0.303% dicalcium phosphate, 1.123% limestone, 0.237% sodium chloride, 0.121% commercial mineral premix (180 g/kg Zinc sulfate , 150 g/kg Copper Sulfate, 10 g / kg Cobalt Sulfate, 10 g / kg sodium selenite and 10 g / kg Potassium Iodate. [*]In spite of distribution from day 4, the starter concentrate intake began from day 8 and calves slaughtered at 28, 49 and 63 days of age consumed mean 188.68 ± SEM 0.01, 265.57±0.01 and 459.99±0.01 g of DMI concentrate/day.

Table S2. Summary of statistical analyses including calf genetic group.

		Alpha diversity - Anova table (Type III tests)											
		Archaea				Bacteria				Fungi			
	**Factor**	**Df[1]**	**Sum sq[2]**	**F[3]**	**Pr(>F)[4]**	**Df**	**Sum sq**	**F**	**Pr(>F)**	**Df**	**Sum sq**	**F**	**Pr(>F)**
**Shannon**	Intercept	1	1.749	15.763	0.000	1	46.224	78.747	0.000	1	7.042	35.104	0.000
	Genetic group	2	0.302	1.362	0.269	2	1.386	1.181	0.318	2	0.063	0.156	0.856
	Diet	1	0.112	1.005	0.323	1	0.027	0.047	0.830	1	0.634	3.158	0.084
	Age	3	0.358	1.076	0.372	3	0.741	0.421	0.739	3	0.591	0.981	0.412
	Residuals	35	3.884			38	22.306			36	7.222		
**Invsimpson**	Intercept	1	20.017	63.209	0.000	1	1016.130	20.367	0.000	1	41.862	28.099	0.000
	Genetic group	2	0.972	1.534	0.230	2	250.380	2.509	0.095	2	0.057	0.019	0.981
	Diet	1	0.412	1.300	0.262	1	6.000	0.120	0.731	1	2.529	1.697	0.201
	Age	3	1.375	1.447	0.246	3	81.680	0.546	0.654	3	1.050	0.235	0.872
	Residuals	35	11.084			38	1895.850			36	53.633		
**Chao1**	Intercept	1	95.588	73.070	0.000	1	153962.000	23.280	0.000	1	240.610	9.030	0.005
	Genetic group	2	2.649	1.012	0.374	2	39262.000	2.968	0.063	2	7.810	0.147	0.864
	Diet	1	0.419	0.321	0.575	1	1.000	0.000	0.989	1	6.330	0.238	0.629
	Age	3	0.876	0.223	0.880	3	7180.000	0.362	0.781	3	25.000	0.313	0.816
	Residuals	35	45.786			38	251317.000			36	959.270		
		**Beta diversity - Permutation test table[5] (Marginal effects a.k.a. Type III tests)**											
**CAP[6]**	Genetic group	2	1.670	0.842	0.530	2	1.136	1.098	0.249	2	1.990	1.316	0.136
	Diet	1	3.646	3.678	0.013	1	1.710	3.305	0.001	1	0.516	0.682	0.839
	Age	3	2.954	0.993	0.420	3	2.329	1.501	0.003	3	2.237	0.987	0.477
	Residuals	35	34.698			38	19.659			36	27.206		

Continued Table S2

	Taxa	Analysis of Deviance table[7] - (*P*-values)		
		Genetic group	Diet	Age
Archaea	*Mbb. gottschalkii* clade	0.123	0.015	0.119
	Mbb. ruminantium clade	0.504	0.013	0.321
	Methanosphaera sp. A4	0.061	0.001	0.160
	Ca. M. alvus	0.123	0.050	0.765
Bacteria	*Acidaminococcus*	0.744	<.001	<.001
	Bacteroides	0.141	<.001	<.001
	Bifidobacterium	0.443	<.001	<.001
	Blautia	0.789	0.026	<.001
	Bulleidia	0.441	<.001	0.097
	Butyricimonas	0.199	0.100	0.005
	Butyrivibrio	0.020	0.566	0.054
	Campylobacter	0.711	<.001	<.001
	CF231	0.595	<.001	<.001
	Clostridium	0.363	0.059	0.043
	Coprococcus	0.950	0.010	0.190
	Corynebacterium	0.545	<.001	<.001
	Desulfovibrio	0.195	0.053	0.053
	Eubacterium	0.572	<.001	<.001
	Faecalibacterium	0.703	<.001	<.001
	Fusobacterium	0.388	0.259	0.051
	Lactobacillus	0.254	<.001	<.001
	Leuconostoc	0.276	0.149	0.034
	Megasphaera	0.883	<.001	<.001
	Odoribacter	0.645	<.001	<.001
	Oribacterium	0.447	<.001	<.001
	Oscillospira	0.331	0.327	<.001
	p-75-a5	0.766	<.001	<.001
	Parabacteroides	0.502	<.001	<.001
	Phascolarctobacterium	0.142	0.080	<.001
	Porphyromonas	0.545	0.068	<.001
	Prevotella	0.222	0.012	0.095
	Pseudoramibacter_Eubacterium	0.950	<.001	<.001
	Ruminococcus	0.438	0.076	0.015
	Sharpea	0.194	<.001	<.001
	SHD.231	0.442	<.001	<.001
	Shuttleworthia	0.206	<.001	<.001
	Streptococcus	0.641	<.001	<.001
	Succiniclasticum	0.522	<.001	0.088
	Succinivibrio	0.944	<.001	<.001
	Synergistes	0.030	0.007	0.024
	Treponema	0.089	0.530	<.001
	YRC22	0.335	0.006	0.124

**Fungi**	*Caecomyces*	0.751	0.309	0.237
	Orpinomyces	0.657	0.019	0.001
	SK3	0.125	0.244	0.111
	f_Neocallimastigaceae	0.129	0.123	0.065

[1]Degrees of freedom*;* [2]Sum of squares; [3]F-test; [4]*P*-values; [5]Permutation test for all constrained eigenvalues by [6]Canonical analysis of principal coordinates using Bray-Curtis dissimilarity index; [7]Differences in the relative abundance of archaeal, bacterial and fungal taxa in response to genetic group, diet and age were assessed by Poisson regression followed by analysis of deviance (F-test, type III error); In all test, *P*-values ≤ 0.05 were considered significant.

Table S3. Summary of chimera identification according to microbial domain, diet and age group.

	Diet[1]	Age[2]	Calves	Sequences prior chimera identification			Chimeras identified		
				**Total**	**Mean**	**SEM[3]**	**Total**	**Mean**	**SEM**
**Archaea**	**M**	**07d**	**6**	37791.000	6298.500	1964.522	456.000	76.000	28.619
		**28d**	**6**	33103.000	5517.167	1903.415	607.000	101.167	58.833
		**49d**	**7**	56027.000	8003.857	2000.781	2502.000	357.429	132.823
		**63d**	**6**	73642.000	12273.667	2616.051	3064.000	510.667	267.353
	**MC**	**28d**	**6**	64193.000	10698.833	2540.987	756.000	126.000	42.498
		**49d**	**8**	37225.000	4653.125	1457.593	1819.000	227.375	112.918
		**63d**	**6**	83117.000	13852.833	5507.014	1361.000	226.833	77.490
**Bacteria**	**M**	**07d**	**6**	55426.000	9237.667	1188.142	2613.000	435.500	151.924
		**28d**	**6**	249342.000	41557.000	12619.624	2164.000	360.667	73.099
		**49d**	**7**	349532.000	49933.143	11491.913	22753.000	3250.429	2613.055
		**63d**	**6**	274136.000	45689.333	7064.888	3261.000	543.500	180.737
	**MC**	**28d**	**6**	86757.000	14459.500	3267.193	4945.000	824.167	214.892
		**49d**	**8**	49575.000	6196.875	1324.577	3075.000	384.375	151.709
		**63d**	**6**	92801.000	15466.833	5181.939	17030.000	2838.333	1354.086
**Fungi**	**M**	**07d**	**5**	108149.000	21629.800	6460.478	33.000	6.600	2.561
		**28d**	**5**	85036.000	17007.200	3849.775	6.000	1.200	0.374
		**49d**	**7**	109549.000	15649.857	3277.707	20.000	2.857	1.262
		**63d**	**6**	147900.000	24650.000	4949.487	36.000	6.000	2.436
	**MC**	**28d**	**6**	100552.000	16758.667	3993.077	30.000	5.000	2.145
		**49d**	**8**	143635.000	17954.375	4226.648	42.000	5.250	2.631
		**63d**	**6**	170112.000	28352.000	4610.857	98.000	16.333	12.244

[1]Calves fed only milk (M) or milk and starter concentrate (MC); [2] slaughter age 7, 28, 49 and 63 days old; [3]Standard error of the mean.

Table S4. Summary of sequencing of archaea, bacteria and fungi from rumen samples of dairy calves according to diet and age group.

				After clean-up								After normalization					
				Sequence			OTU			Coverage		Sequence			OTU		
	Diet[1]	Age[2]	Calves	Total	Mean	SEM[3]	Total	Mean	SEM	Mean	SEM	Total	Mean	SEM	Total	Mean	SEM
Archaea	M	07d	6	7554.000	1259.000	674.486	22.000	3.667	0.843	1.000	0.000	617.000	102.833	0.167	16.000	2.667	0.494
		28d	6	13045.000	2174.167	787.831	25.000	4.167	0.601	0.999	0.001	618.000	103.000	0.000	19.000	3.167	0.401
		49d	7	10099.000	1442.714	553.438	25.000	3.571	0.481	1.000	0.000	720.000	102.857	0.261	24.000	3.429	0.481
		63d	6	11489.000	1914.833	794.196	27.000	4.500	0.500	1.000	0.000	616.000	102.667	0.333	22.000	3.667	0.494
	MC	28d	6	13410.000	2235.000	919.420	25.000	4.167	0.307	1.000	0.000	616.000	102.667	0.333	21.000	3.500	0.428
		49d	8	9141.000	1142.625	535.473	28.000	3.500	0.423	1.000	0.000	823.000	102.875	0.125	25.000	3.125	0.398
		63d	6	19542.000	3257.000	1622.311	24.000	4.000	0.365	1.000	0.000	617.000	102.833	0.167	19.000	3.167	0.401
Bacteria	M	07d	6	41499.000	6916.500	994.973	687.000	114.500	8.625	0.998	0.000	18089.000	3014.833	3.135	642.000	107.000	10.040
		28d	6	217861.000	36310.167	11456.553	1008.000	168.000	3.077	0.998	0.001	18056.000	3009.333	5.463	670.000	111.667	16.808
		49d	7	282921.000	40417.286	9475.404	1880.000	268.571	48.987	0.999	0.000	20940.000	2991.429	5.830	901.000	128.714	16.643
		63d	6	240662.000	40110.333	6380.079	1063.000	177.167	7.993	0.999	0.000	18017.000	3002.833	3.371	596.000	99.333	9.566
	MC	28d	6	64378.000	10729.667	2710.992	896.000	149.333	28.393	0.997	0.001	18047.000	3007.833	4.430	712.000	118.667	22.921
		49d	8	72065.000	9008.125	2520.070	895.000	111.875	12.710	0.997	0.001	24095.000	3011.875	2.682	813.000	101.625	15.565
		63d	6	60497.000	10082.833	3478.488	969.000	161.500	33.798	0.997	0.001	18069.000	3011.500	8.702	727.000	121.167	21.642
Fungi	M	07d	5	7795.000	1559.000	556.267	60.000	12.000	1.265	0.999	0.001	529.000	105.800	0.490	33.000	6.600	0.980
		28d	5	2055.000	411.000	153.557	56.000	11.200	1.908	0.988	0.006	529.000	105.800	0.200	42.000	8.400	0.927
		49d	7	1502.000	214.571	68.444	63.000	9.000	1.431	0.989	0.004	743.000	106.143	0.459	50.000	7.143	0.986
		63d	6	2903.000	483.833	279.104	61.000	10.167	2.372	0.987	0.005	643.000	107.167	0.543	42.000	7.000	1.000
	MC	28d	6	3906.000	651.000	272.819	59.000	9.833	1.447	0.995	0.003	641.000	106.833	0.543	42.000	7.000	0.931
		49d	8	8108.000	1013.500	449.988	66.000	8.250	1.840	0.997	0.002	852.000	106.500	0.327	41.000	5.125	1.060
		63d	6	5830.000	971.667	442.993	95.000	15.833	3.439	0.994	0.002	635.000	105.833	0.477	45.000	7.500	0.764

[1]Calves fed only milk (M) or milk and starter concentrate (MC); [2] slaughter age 7, 28, 49 and 63 days old; [3]Standard error of the mean.

Table S5. Alpha-diversity of archaeal, bacterial and fungal communities in the rumen of calves according diet, age groups.

		**Diet[1]**			
	**Index**	**M**	**MC**	***P*-value[*]**	**FDR**
**Arc**	Chao1	3.320±1.180	3.300±1.129	0.465	0.608
	Invsimpson	1.640±0.614	1.475±0.516	0.109	0.328
	Shannon	0.571±0.369	0.482±0.331	0.198	0.476
**Bac**	Chao1	165.564±72.734	173.746±94.046	0.219	0.490
	Invsimpson	11.704±7.322	12.431±7.151	0.157	0.472
	Shannon	2.915±0.690	2.954±0.832	0.391	0.496
**Fun**	Chao1	9.036±4.413	8.588±5.444	0.741	0.967
	Invsimpson	2.952±1.125	2.371±1.19	0.709	0.967
	Shannon	1.228±0.367	0.951±0.508	0.775	0.967

		**Age[2]**					
		**07d**	**28d**	**49d**	**63d**		
**Arc**	Chao1	2.667±1.211	3.333±0.985	3.333±1.175	3.583±1.240	0.345	0.608
	Invsimpson	1.419±0.504	1.702±0.557	1.423±0.494	1.686±0.705	0.390	0.608
	Shannon	0.432±0.340	0.634±0.313	0.445±0.378	0.587±0.362	0.507	0.608
**Bac**	Chao1	143.93±28.079	166.602±91.008	176.645±87.304	175.128±89.866	0.641	0.672
	Invsimpson	12.193±6.712	14.238±7.492	11.839±7.214	9.97±7.279	0.322	0.490
	Shannon	2.975±0.686	3.138±0.666	2.88±0.885	2.772±0.709	0.672	0.672
**Fun**	Chao1	7.500±3.202	8.909±3.846	8.500±5.441	9.715±5.803	0.847	0.967
	Invsimpson	3.171±0.914	2.665±1.018	2.446±1.347	2.788±1.246	0.896	0.967
	Shannon	1.294±0.342	1.185±0.310	0.923±0.610	1.159±0.340	0.967	0.967

		**Diet*Age[3]**								
		**M_07d**	**M_28d**	**M_49d**	**M_63d**	**MC_28d**	**MC_49d**	**MC_63d**		
**Arc**	Chao1	2.667±1.211	3.167±0.983	3.571±1.272	3.833±1.169	3.5±1.0490	3.125±1.126	3.333±1.366	0.618	0.646
	Invsimpson	1.419±0.504	1.689±0.440	1.516±0.547	1.958±0.892	1.716±0.699	1.341±0.464	1.414±0.345	0.473	0.608
	Shannon	0.432±0.340	0.632±0.321	0.509±0.384	0.722±0.445	0.635±0.337	0.39±0.390	0.452±0.218	0.646	0.646
**Bac**	Chao1	143.93±28.079	172.835±109.299	195.941±84.597	144.488±40.254	160.37±78.625	159.761±91.715	205.768±117.871	0.327	0.490
	Invsimpson	12.193±6.712	14.185±8.793	13.246±8.176	6.937±4.059	14.29±6.793	10.607±6.563	13.003±8.832	0.309	0.490
	Shannon	2.975±0.686	3.012±0.825	3.07±0.778	2.578±0.468	3.263±0.507	2.714±0.989	2.965±0.893	0.413	0.496
**Fun**	Chao1	7.500±3.202	10.400±4.602	9.000±5.627	9.222±4.277	7.667±2.927	8.062±5.622	10.208±7.43	0.689	0.967
	Invsimpson	3.171±0.914	2.628±1.460	3.057±1.192	2.917±1.148	2.697±0.605	1.912±1.308	2.659±1.434	0.408	0.967
	Shannon	1.294±0.342	1.172±0.452	1.249±0.432	1.195±0.320	1.196±0.168	0.638±0.621	1.124±0.386	0.128	0.385

Values represent mean and standard deviation. [1]milk (M), milk and concentrate (MC); [2]slaughter age (7, 28, 49 and 63 days); [3]Calves fed only with milk (M) or milk and starter concentrate (MC) that were slaughtered at 7, 28, 49 and 63 days of age;[*]*P*-value adjusted by FDR method; FDR≤ 0.05 were considered significant.

Table S6. Mean abundances of bacterial genera which were not different (FDR>0.05) between diet, age or interaction (diet*age) groups.

	Diet[1]					
**Genus**	**M**		**MC**		***P*-value**	**FDR[*]**
Butyricimonas	0.218±0.086		0.055±0.023		0.163	0.215
Butyrivibrio	1.793±0.708		2.153±0.696		0.651	0.720
Clostridium	0.083±0.039		0.058±0.023		0.098	0.140
Coprococcus	0.441±0.123		0.207±0.104		0.037	0.060
Desulfovibrio	0.286±0.090		0.289±0.056		0.626	0.700
Fusobacterium	0.232±0.080		0.089±0.057		0.291	0.353
Leuconostoc	0.101±0.064		0.075±0.061		0.196	0.250
Oscillospira	0.706±0.245		0.464±0.139		0.418	0.486
Phascolarctobacterium	0.158±0.065		0.073±0.053		0.823	0.860
Porphyromonas	1.105±0.452		0.540±0.529		0.688	0.740
Prevotella	9.677±2.424		16.817±4.144		0.223	0.276
Ruminococcus	5.136±0.949		8.081±2.238		0.889	0.897
Treponema	0.121±0.071		0.245±0.135		0.423	0.487
	Age[2]					
	**07d**	**28d**	**49d**	**63d**		
Bulleidia	0.327±0.327	1.127±0.387	1.32±0.432	1.292±0.668	0.207	0.259
Butyrivibrio	0.066±0.043	2.368±0.826	1.675±0.669	2.830±1.426	0.090	0.130
Clostridium	0.006±0.006	0.028±0.015	0.102±0.058	0.111±0.047	0.051	0.079
Coprococcus	0.072±0.054	0.307±0.189	0.36±0.136	0.471±0.182	0.319	0.379
Desulfovibrio	0.519±0.304	0.271±0.074	0.162±0.049	0.343±0.104	0.147	0.200
Fusobacterium	0.441±0.220	0.225±0.118	0.069±0.042	0.102±0.087	0.101	0.142
Leuconostoc	0.000±0.000	0.022±0.014	0.237±0.126	0.017±0.014	0.064	0.095
Prevotella	9.118±3.987	12.196±2.941	14.054±5.227	13.865±4.878	0.406	0.477
Ruminococcus	2.180±1.040	4.389±1.465	8.259±2.780	8.365±1.624	0.047	0.073
Succiniclasticum	0.986±0.986	1.829±0.801	1.310±0.682	0.956±0.387	0.847	0.870
YRC22	0.110±0.07	0.030±0.027	0.042±0.029	0.088±0.054	0.206	0.259

Continued Table S6

	Diet*Age[3]								
	**M-07d**	**M-28d**	**M-49d**	**M-63d**	**MC-28d**	**MC-49d**	**MC-63d**		
Bifidobacterium	0.745±0.574	0.250±0.250	0.000±0.000	0.000±0.000	8.239±3.732	0.728±0.645	0.216±0.183	0.038	0.061
Bulleidia	0.327±0.327	0.222±0.183	0.568±0.503	0.050±0.032	2.033±0.545	1.977±0.614	2.533±1.160	0.183	0.237
Butyricimonas	0.508±0.233	0.225±0.194	0.149±0.143	0.000±0.000	0.066±0.026	0.021±0.012	0.088±0.070	0.110	0.153
Butyrivibrio	0.066±0.043	2.167±1.232	1.082±0.370	3.977±2.574	2.569±1.210	2.193±1.223	1.683±1.342	0.479	0.541
Clostridium	0.006±0.006	0.022±0.022	0.181±0.12	0.106±0.073	0.033±0.023	0.033±0.025	0.116±0.065	0.137	0.188
Coprococcus	0.072±0.054	0.608±0.348	0.596±0.232	0.461±0.253	0.006±0.006	0.153±0.126	0.480±0.287	0.175	0.230
Desulfovibrio	0.519±0.304	0.194±0.107	0.100±0.054	0.361±0.178	0.349±0.102	0.216±0.078	0.326±0.127	0.663	0.726
Eubacterium	0.485±0.445	0.011±0.007	0.010±0.006	0.011±0.007	0.050±0.032	0.121±0.107	0.116±0.041	0.844	0.870
Faecalibacterium	0.000±0.000	0.006±0.006	0.149±0.143	0.000±0.000	0.028±0.010	0.472±0.462	0.033±0.027	0.066	0.096
Fusobacterium	0.441±0.220	0.427±0.211	0.067±0.056	0.022±0.007	0.022±0.016	0.070±0.066	0.182±0.175	0.064	0.095
Leuconostoc	0.000±0.000	0.017±0.011	0.324±0.215	0.028±0.028	0.028±0.028	0.162±0.152	0.006±0.006	0.856	0.871
Oscillospira	2.009±0.822	0.337±0.153	0.263±0.169	0.288±0.132	0.631±0.141	0.224±0.111	0.617±0.422	0.446	0.508
Phascolarctobacterium	0.503±0.218	0.061±0.020	0.081±0.053	0.000±0.000	0.017±0.011	0.041±0.041	0.170±0.170	0.161	0.215
Prevotella	9.118±3.987	8.490±3.281	6.676±2.876	14.923±8.447	15.903±4.668	20.509±9.138	12.808±5.737	0.304	0.365
Pseudoramibacter	0.022±0.011	0.028±0.018	0.024±0.019	0.017±0.011	0.072±0.029	0.290±0.162	0.338±0.123	0.058	0.089
Ruminococcus	2.180±1.040	3.676±1.815	5.631±2.136	8.974±1.435	5.101±2.438	10.558±4.892	7.757±3.064	0.755	0.797
SHD.231	0.000±0.000	0.000±0.000	0.647±0.625	0.522±0.515	0.000±0.000	0.000±0.000	0.000±0.000	1.000	1.000
Succiniclasticum	0.986±0.986	0.116±0.055	0.586±0.376	0.144±0.077	3.541±1.284	1.944±1.230	1.767±0.623	0.260	0.319
Treponema	0.000±0.000	0.454±0.265	0.019±0.019	0.028±0.010	0.491±0.421	0.087±0.058	0.209±0.163	0.669	0.726
YRC22	0.110±0.070	0.005±0.005	0.000±0.000	0.028±0.028	0.055±0.055	0.079±0.052	0.149±0.103	0.738	0.786

Values represent mean and standard deviation. [1]milk (M), milk plus starter concentrate (MC); [2]slaughter age (7, 28, 49 and 63 days); [3]Calves fed with only milk (M) or milk plus starter concentrate (MC) that were slaughtered at 7, 28, 49 and 63 days of age; [*]*P*-value adjusted by FDR method; FDR≤ 0.05 were considered significant.

Table S7. Co-occurrence (Dice index) and correlation (Spearman´s rank) analysis of inter-intra microbial taxa and between relative abundance of archaeal, bacterial and fungal taxa and molar proportions of volatile fatty acids in rumen samples of calves grouped according to diet. Only significant (*P*-value ≤0.05) correlations (Spearman>0.6 or <0.6) are show.

**Diet[1]**	**Association[2]**	**Dice**	**Spearman**	***P*-value[3]**
	arc|s_Mbb. gottschalkii clade*arc|s_Ca. Methanomethylophilus alvus	0.39	-0.76	<.001
	arc|s_Methanosphaera sp. A4*arc|s_Mbb. gottschalkii clade	0.50	-0.70	<.001
	bac|f_S24-7*bac|f_Coriobacteriaceae	0.84	0.84	<.001
	bac|g_Acidaminococcus*bac|f_Veillonellaceae	0.73	0.74	<.001
	bac|g_Blautia*bac|f_Neisseriaceae	0.91	0.60	0.001
	bac|g_Bulleidia*bac|f_Veillonellaceae	0.78	0.69	<.001
	bac|g_Butyrivibrio*bac|g_Bacteroides	0.86	-0.67	<.001
	bac|g_Lactobacillus*bac|g_Acidaminococcus	0.28	-0.82	<.001
	bac|g_Parabacteroides*bac|g_Bacteroides	0.88	0.64	0.001
	bac|g_Paraprevotella*bac|f_S24-7	0.73	0.63	0.001
	bac|g_Paraprevotella*bac|g_Oscillospira	0.79	0.75	<.001
	bac|g_Prevotella*bac|f_Paraprevotellaceae	0.79	0.72	<.001
	bac|g_Prevotella*bac|f_S24-7	0.56	0.64	0.001
	bac|g_Ruminococcus*bac|g_Bacteroides	0.94	-0.76	<.001
	bac|g_Ruminococcus*bac|g_Blautia	0.89	-0.64	0.001
	bac|g_Shuttleworthia*bac|g_Bacteroides	0.68	-0.66	<.001
	bac|g_Streptococcus*bac|f_Neisseriaceae	0.85	0.66	<.001
	bac|g_Streptococcus*bac|g_Bacteroides	0.81	0.62	0.001
**M**	bac|g_Streptococcus*bac|g_Blautia	0.85	0.74	<.001
	bac|g_Streptococcus*bac|g_Ruminococcus	0.78	-0.66	<.001
	bac|g_Streptococcus*bac|g_Shuttleworthia	0.50	-0.71	<.001
	bac|g_Streptococcus*bac|p_Firmicutes	0.84	-0.63	0.001
	bac|g_Succiniclasticum*bac|f_Coriobacteriaceae	0.64	0.62	0.001
	bac|g_Succiniclasticum*bac|f_S24-7	0.67	0.61	0.001
	bac|g_Succiniclasticum*bac|f_Veillonellaceae	0.83	0.78	<.001
	bac|g_Succiniclasticum*bac|g_Acidaminococcus	0.73	0.61	0.001
	bac|g_Succiniclasticum*bac|g_Bulleidia	0.87	0.88	<.001
	bac|g_Succiniclasticum*bac|g_Megasphaera	0.63	0.61	0.001
	bac|g_Succinivibrio*bac|f_Veillonellaceae	0.80	0.80	<.001
	bac|g_Succinivibrio*bac|g_Acidaminococcus	0.67	0.67	<.001
	bac|g_Succinivibrio*bac|g_Bulleidia	0.74	0.66	<.001
	bac|g_Succinivibrio*bac|g_Succiniclasticum	0.80	0.66	<.001
	bac|s_Prevotella ruminicola*bac|g_Ruminococcus	0.93	0.70	<.001
	bac|s_Ruminococcus bromii*bac|f_Mogibacteriaceae	0.76	0.60	0.001
	bac|s_Ruminococcus bromii*bac|f_Veillonellaceae	0.77	0.65	<.001
	bac|s_Ruminococcus bromii*bac|g_Ruminococcus	0.76	0.68	<.001
	bac|s_Ruminococcus flavefaciens*bac|g_Butyrivibrio	0.88	0.62	0.001

	bac|s_Ruminococcus flavefaciens*bac|p_Firmicutes	0.91	0.62	0.001
	fun|s_Caecomyces 1*arc|s_Methanosphaera sp. A4	0.30	-0.61	0.001
	Acetate:Propionate*Acetate	1.00	0.65	<.001
	Propionate*AP	1.00	-0.84	<.001
	Total VFA*arc|s_Ca. Methanomethylophilus alvus	0.68	-0.68	<.001
	Total VFA*bac|g_Butyrivibrio	0.89	0.68	<.001
	Total VFA*bac|g_Streptococcus	0.84	-0.61	0.001
**MC**	arc|s_Methanosphaera sp. A4*arc|s_Mbb. gottschalkii clade	0.33	-0.76	<.001
	arc|s_Methanosphaera sp. A4*arc|s_Mbb. ruminantium clade	0.40	-0.71	<.001
	bac|f_Mogibacteriaceae*bac|f_Coriobacteriaceae	0.97	0.61	0.004
	bac|f_Ruminococcaceae*arc|s_Methanosphaera sp. A4	0.79	-0.62	0.004
	bac|f_S24-7*bac|f_Coriobacteriaceae	1.00	0.60	0.005
	bac|f_Veillonellaceae*bac|f_S24-7	0.94	0.67	0.001
	bac|g_Acidaminococcus*bac|f_Mogibacteriaceae	0.82	0.70	0.001
	bac|g_Acidaminococcus*bac|f_Ruminococcaceae	0.86	-0.63	0.003
	bac|g_Acidaminococcus*bac|f_Veillonellaceae	0.90	0.73	<.001
	bac|g_Bulleidia*bac|f_S24-7	0.91	0.66	0.002
	bac|g_Bulleidia*bac|f_Veillonellaceae	0.85	0.68	0.001
	bac|g_Lactobacillus*bac|f_Ruminococcaceae	0.62	0.61	0.004
	bac|g_Lactobacillus*bac|g_Blautia	0.70	0.62	0.003
	bac|g_Megasphaera*arc|s_Ca. Methanomethylophilus alvus	0.81	0.75	<.001
	bac|g_Megasphaera*bac|f_Coriobacteriaceae	0.91	0.61	0.005
	bac|g_Megasphaera*bac|f_Mogibacteriaceae	0.88	0.60	0.005
	bac|g_Megasphaera*bac|f_S24-7	0.91	0.62	0.004
	bac|g_Megasphaera*bac|f_Veillonellaceae	0.90	0.71	0.001
	bac|g_Parabacteroides*bac|f_Neisseriaceae	0.75	0.67	0.001
	bac|g_Paraprevotella*arc|s_Mbb. gottschalkii clade	0.73	0.78	<.001
	bac|g_Paraprevotella*bac|g_Bacteroides	0.86	0.63	0.003
	bac|g_Prevotella*bac|f_S24-7	0.95	0.61	0.004
	bac|g_Prevotella*bac|f_Veillonellaceae	0.89	0.60	0.005
	bac|g_Streptococcus*bac|f_Neisseriaceae	0.82	0.74	<.001
	bac|g_Streptococcus*bac|g_Parabacteroides	0.80	0.67	0.001
	bac|g_Succiniclasticum*bac|f_Veillonellaceae	0.94	0.75	<.001
	bac|g_Succiniclasticum*bac|g_Bacteroides	0.82	-0.64	0.002
	bac|p_Bacteroidetes*arc|s_Methanosphaera sp. A4	0.75	-0.60	0.005
	bac|s_Prevotella ruminicola*bac|g_Succinivibrio	0.83	0.69	0.001
	bac|s_Ruminococcus flavefaciens*bac|g_Ruminococcus	0.92	0.61	0.004
	Acetate:Propionate*Acetate	1.00	0.83	<.001
	Propionate*bac|s_Ruminococcus bromii	0.71	0.70	0.001

[1]Calves fed with whole milk (M) or whole milk and starter concentrate (MC); [2]arc (archaea); bac (bacteria), fun (fungi); [3]Significance of Spearman´s correlation.

Table S8. Volatile fatty acids (VFA) concentration in the rumen samples of calves according diet and age group.

	**Diet[1]**			
**VFA (mmol/L)**	**M**	**MC**	***P*-value**	**FDR[*]**
Acetate:Propionate	3.492 ± 1.554	3.288 ± 1.973	0.777	0.829
Acetate	12.523 ± 4.601 [b]	35.443 ± 12.532 [a]	<.001	<.001
Butyrate	2.200 ± 1.733 [b]	5.829 ± 4.552 [a]	<.001	<.001
Isobutyrate	0.931 ± 1.101 [b]	1.917 ± 1.281 [a]	0.021	0.049
Isovalerate	0.713 ± 0.576 [b]	1.878 ± 1.267 [a]	<.001	<.001
Propionate	4.427 ± 2.847 [b]	13.252 ± 7.621 [a]	<.001	<.001
Valerate	0.693 ± 0.978 [b]	2.287 ± 1.943 [a]	<.001	0.001
Total VFA	46.066 ± 21.06 [b]	124.879 ± 57.095 [a]	<.001	<.001

	**Age[2]**					
	**07d**	**28d**	**49d**	**63d**		
Acetate:Propionate	3.487 ± 0.813	3.136 ± 1.354	3.961 ± 2.511	2.925 ± 1.059	0.714	0.816
Acetate	6.168 ± 1.500[d]	17.172 ± 6.344[c]	24.103 ± 10.228[b]	34.776 ± 18.153[a]	<.001	<.001
Butyrate	0.938 ± 0.536	3.251 ± 2.108	3.514 ± 3.059	6.186 ± 5.295	0.682	0.808
Isobutyrate	0.212 ± 0.122	1.101 ± 0.473	1.666 ± 1.642	1.845 ± 1.274	0.341	0.474
Isovalerate	0.097 ± 0.15	1.257 ± 0.657	1.163 ± 0.706	1.857 ± 1.629	0.113	0.191
Propionate	1.875 ± 0.645	6.424 ± 3.259	8.102 ± 5.104	13.82 ± 9.646	0.312	0.453
Valerate	0.065 ± 0.159	1.347 ± 1.202	1.251 ± 1.161	2.312 ± 2.468	0.585	0.748
Total VFA	20.313 ± 5.329	65.717 ± 21.788	80.397 ± 36.644	127.733 ± 77.648	0.043	0.081

	**Diet*Age[3]**								
	**M_07d**	**M_28d**	**M_49d**	**M_63d**	**MC_28d**	**MC_49d**	**MC_63d**		
Acetate:Propionate	3.487±0.813	3.177±1.651	4.124±2.278	3.073±1.042	3.817±1.848	3.095±1.142	2.777±1.152	0.985	0.985
Acetate	6.168±1.500[f]	11.213±0.516[ef]	14.213±1.882[de]	18.217±1.202[cd]	23.132±1.749[c]	32.758±4.771[b]	51.335±8.088[a]	<.001	<.001
Butyrate	0.938±0.536[b]	2.703±2.098[b]	2.506±2.108[b]	2.602±1.363[b]	3.798±2.158[b]	4.396±3.606[b]	9.77±5.385[a]	0.027	0.035
Isobutyrate	0.212±0.122	1.040±0.539	1.347±1.863	1.057±0.671	1.162±0.439	1.945±1.492	2.633±1.275	0.290	0.441
Isovalerate	0.097±0.150[c]	1.187±0.798[bc]	0.877±0.180[bc]	0.665±0.356[bc]	1.327±0.547[bc]	1.413±0.903[b]	3.05±1.517[a]	0.002	0.006
Propionate	1.875±0.645[c]	4.613±2.913[c]	4.700±3.273[c]	6.475±2.038[bc]	8.235±2.647[bc]	13.079±4.607[b]	21.165±8.429[a]	0.007	0.016
Valerate	0.065±0.159[b]	1.102±1.444[b]	0.856±0.958[b]	0.723±0.818[b]	1.592±0.975[b]	1.596±1.271[b]	3.902±2.582[a]	0.017	0.033
TotalVFA	20.313±5.329[d]	50.865±17.63[cd]	49.009±18.403[cd]	63.587±13.389[cd]	80.568±14.289[bc]	107.861±23.408[b]	191.88±56.654[a]	<.001	<.001

Values represent mean and standard deviation. [1]milk (M), milk plus concentrate (MC); [2]slaughter age (7, 28, 49 and 63 days); [3]Calves fed with only with milk (M) or milk plus starter concentrate (MC) that were slaughtered at 7, 28, 49 and 63 days of age; [*]*P*-value adjusted by FDR method; FDR ≤ 0.05 were considered significant.
